# Human Milk Oligosaccharides: Decoding Their Structural Variability, Health Benefits, and the Evolution of Infant Nutrition

**DOI:** 10.3390/nu17010118

**Published:** 2024-12-30

**Authors:** Hatice Duman, Mikhael Bechelany, Sercan Karav

**Affiliations:** 1 Department of Molecular Biology and Genetics, Çanakkale Onsekiz Mart University, Çanakkale 17100, Türkiye; hatice.duman@comu.edu.tr; 2 Institut Européen des Membranes (IEM), UMR 5635, University Montpellier, ENSCM, CNRS, F-34095 Montpellier, France; 3 Functional Materials Group, Gulf University for Science and Technology (GUST), Masjid Al Aqsa Street, Mubarak Al-Abdullah 32093, Kuwait

**Keywords:** human milk oligosaccharides, breastfeeding, prebiotics, nutrition, *Bifidobacteria*, synthesis, glycoside hydrolases, infant formula, health benefits

## Abstract

Human milk oligosaccharides (HMOs), the third most abundant solid component in human milk, vary significantly among women due to factors such as secretor status, race, geography, season, maternal nutrition and weight, gestational age, and delivery method. In recent studies, HMOs have been shown to have a variety of functional roles in the development of infants. Because HMOs are not digested by infants, they act as metabolic substrates for certain bacteria, helping to establish the infant’s gut microbiota. By encouraging the growth of advantageous intestinal bacteria, these sugars function as prebiotics and produce short-chain fatty acids (SCFAs), which are essential for gut health. HMOs can also specifically reduce harmful microbes and viruses binding to the gut epithelium, preventing illness. HMO addition to infant formula is safe and promotes healthy development, infection prevention, and microbiota. Current infant formulas frequently contain oligosaccharides (OSs) that differ structurally from those found in human milk, making it unlikely that they would reproduce the unique effects of HMOs. However, there is a growing trend in producing OSs resembling HMOs, but limited data make it unclear whether HMOs offer additional therapeutic benefits compared to non-human OSs. Better knowledge of how the human mammary gland synthesizes HMOs could direct the development of technologies that yield a broad variety of complex HMOs with OS compositions that closely mimic human milk. This review explores HMOs’ complex nature and vital role in infant health, examining maternal variation in HMO composition and its contributing factors. It highlights recent technological advances enabling large-scale studies on HMO composition and its effects on infant health. Furthermore, HMOs’ multifunctional roles in biological processes such as infection prevention, brain development, and gut microbiota and immune response regulation are investigated. The structural distinctions between HMOs and other mammalian OSs in infant formulas are discussed, with a focus on the trend toward producing more precise replicas of HMOs found in human milk.

## 1. Introduction

Breast milk is the solely recommended form of sustenance for infants. It not only supports newborn growth but also contains bioactive components that promote age-appropriate development and immunity. The World Health Organization (WHO) and pediatric societies advise initiating breastfeeding within the first hour after birth and recommend exclusive breastfeeding for the first six months, with continued breastfeeding for up to two years [1,2].

While enhancing our knowledge of the health advantages of breastfeeding, recent developments in analytical methods and the creation and integration of “-omics” technology have provided insightful information about the composition of breast milk [3]. The vast majority of breast milk is composed of water in addition to a wide range of solid components that include both nutritional and bioactive properties. Human milk oligosaccharides (HMOs) are a significantly represented group among the bioactive components of breast milk, both in terms of quantities and structural variety. Scientists have been researching HMOs for over a century, motivated by compelling evidence of mother’s milk’s nutritional and physiological benefits [4]. HMOs were initially “discovered” at the end of the nineteenth century when French biochemist Georges Denigés observed that human and bovine milk contained a number of additional carbohydrate structures in addition to lactose (Lac). Polonowski and Lespagnol initially referred to these unidentified fractions as gynolactoses [5].

Over 200 HMO structures have been identified owing to significant advancements in the science of glycomics, and human milk currently contains 100–1000 times more oligosaccharides (OSs) than any other milk from a domesticated farm animal [6,7]. One liter of mature human milk contains 10–15 g of HMOs, a quantity that often surpasses the total protein content and is 100 to 1000 times greater than the concentration of OSs found in bovine milk, which is commonly used in infant formulas. Human colostrum contains even higher concentrations of HMOs [8].

In the process of producing HMOs, the initial step involves the enzymatic elongation of Lac through either β1–3 or β1–6 linkages of Gal to lacto-*N*-biose (LNB) or *N*-acetyllactosamine (LacNAc). Based on this, HMOs can be classified into either Type-I or Type-II chains. Lacto-*N*-tetraose (LNT), connected to LNB, is contained inside type-I chain HMOs. Type-II chains, on the other hand, are composed of the lacto-*N*-neotetraose (LNnT) isomer of lacto-*N*-neotetraose and possess the ability to bind Lac to LacNAc [8,9]. Additionally, Sia could decorate the core HMO structures with α2–3 or α2–6 linkages, while Fuc can do the same with α1–2, α1–3, or α1–4 linkages at the terminal locations. As a result, HMO may be broadly divided into three categories: neutral OS, sialylated OS, and fucosylated OS (FucOS) [8].

*Bacteroides*, *Lactobacillus*, and *Bifidobacterium* species make up the majority of the gut microbiota of a breast-fed newborn. This is due to the fact that these strains are able to metabolize HMOs [10]. The majority of *Bifidobacterium* species can metabolize the primary HMOs found in breast milk, such as LNT and 2′-fucosyllactose (2′-FL) [11]. Enzymes such as β-galactosidase, α-fucosidase, and sialidase are used by *Bifidobacterium* and *Bacteroides* species, particularly *B. bifidum* and *B. fragilis*, to hydrolyze HMOs [10]. Breast-fed infants have a high concentration of probiotic *Bifidobacterium* species, such as *Bacteroides fragilis* (*B. fragilis*), *Bacteroides vulgatus* (*B. vulgatus*), and *Bifidobacterterium longum* subsp. *Infantis* (*B. infantis*), which operates by this method [12].

HMOs, as essential bioactive constituents, serve as prebiotics, mucosal signaling agents, and immunomodulators, significantly contributing to the enrichment of gut microbiota, the enhancement of intestinal epithelial barrier integrity, and the support of immune function [13]. One of HMOs’ main roles is to act as prebiotics, which helps the infant’s gut microbiota develop healthy bacteria. Therefore, a limited number of dominant beneficial species that inhibit the development of harmful bacteria are selected for by HMOs. As a result, breast-fed and formula-fed infants have quite different microbial compositions in their guts. Formula-fed infants have a more diverse microbiome because formula does not mimic the makeup of breast milk and does not include HMOs [14,15]. Kuhn and György discovered in 1954 that a mix of HMOs, which they called the “bifidus factor”, encourages the growth of *Bifidobacterium bifidum* (*B. bifidum*), which was formerly known as *Lactobacillus bifidus*. The fermentation of HMOs by *Bifidobacterium* produces short-chain fatty acids (SCFAs; butyrate, propionate, and acetate). SCFAs manage gut pH and strengthen the immune system, protecting the commensal flora from invasive infections and inflammation [16,17]. Besides functioning as prebiotics, HMOs have bacteriostatic and antibiofilm characteristics. Preliminary research indicated that both homogeneous and heterogeneous HMOs regulate bacterial proliferation and biofilm formation [18].

Incorporating HMO supplements into newborn formula is safe, promotes healthy growth, and has advantages for the microbiota and infection prevention. It is doubtful that infant formulas would mimic the special effects of HMOs since they frequently contain OSs that are different from those in human milk. However, producing OSs that resemble human milk is becoming more and more popular. It is unknown if HMOs have any therapeutic benefits because there are not enough data to compare their effects with non-human OSs. Comprehending the synthesis of human mammary glands may help direct the creation of intricate HMOs [19].

The present article examines the extensive and intricate characteristics of HMOs and their essential contribution to neonatal health. The significant variability in HMO composition among mothers and the influencing variables were examined. Recent technological advancements facilitating extensive research on HMO makeup and its effects on infant health outcomes were emphasized. Moreover, the multifunctional roles of HMOs in biological processes, including infection prevention, brain development, and the control of gut microbiota and immune response, were examined. The structural differences between HMOs and other mammalian OSs in newborn formulas were examined, emphasizing the tendency toward creating more accurate reproductions of HMOs present in human milk.

## 2. HMOs and Factors Influencing Their Composition in Breast Milk

HMOs rank as the third most significant solid constituent in human milk, following Lac and lipids. The current identification of over 200 structurally distinct HMOs has been discovered. HMOs exhibit resistance to pancreatic and brush border enzymes as well as low stomach pH. Most are metabolized by the gut bacteria of infants or excreted unaltered, with 1 to 2% absorbed, entering the systemic circulation, and being expelled by urine. Moreover, HMOs are resistant to both high and low temperatures, making them impervious to pasteurization and freeze drying [20].

**Figure 1:**
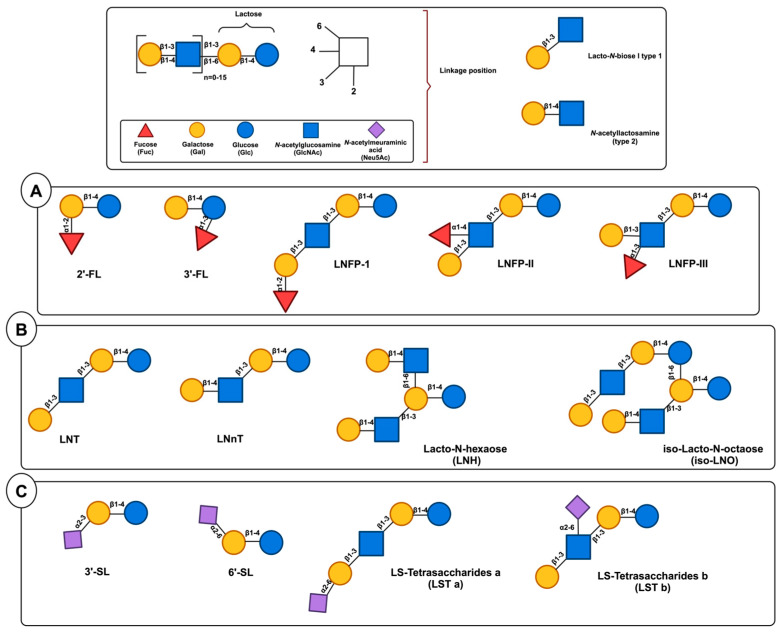
Classification of most common HMOs: (**A**) Representative fucosylated neutral HMOs; (**B**) representative non-fucosylated neutral HMOs; (**C**) representative sialylated HMOs [18,24].

HMOs are a heterogeneous collection of intricate glycans consisting of five primary monosaccharides including galactose (Gal), glucose (Glc), *N*-acetylglucosamine (GlcNAc), fucose (Fuc), and the sialic acid (Sia) derivative *N*-acetylneuraminic acid (Neu5Ac), whose complexity is attributed to the variety of their glycosidic connections. All HMOs include Lac (Galβ1–4Glc) at the reducing end. This Lac can be extended in β1–3- or β1–6-linkage by two distinct disaccharides, namely, Galβ1-3GlcNAc (type 1 chain) or Galβ1–4GlcNAc (type 2 chain). HMOs can be divided into a few distinct classes according to the chemical makeup of their constituent parts [21,22]. Acidic HMOs that include one or more Sia are distinguished from neutral HMOs, which is the fundamental distinction that can be made within this group. The latter can be further delineated into those that include one or more Fuc units against those that lack a Fuc component. One can further divide the acidic sialylated HMOs into acidic non-fucosylated HMOs and acidic fucosylated HMOs [4]. Based on this classification, research indicates that the human milk of secretor women consists of three main groups of HMOs: neutral fucosylated HMOs (35–50%), acidic sialylated HMOs (12–14%), and neutral non-fucosylated HMOs (42–55%) (Figure 1) [23].

In colostrum, the concentration of HMOs ranges from 20 to 25 g/L (with an average of 9–22 g/L), whereas in mature milk, the concentration ranges from 10 to 15 g/L (with an average of 8–19 g/L), and after six months, it ranges from 4 to 6 g/L [25]. The concentration and composition of human milk’s HMOs exhibit variation due to both genetic and non-genetic causes. The fundamental component that contributes to this variety is the activity of two enzymes, namely, Secretor (Se) and Lewis (Le), which are generated by the Se and Le genes in the mother. These genes are responsible for the formation of the enzymes. It is the Se and Le genes that are accountable for the encoding of the enzymes α1–2-fucosyltransferase (FUT2) and α1–3/4-fucosyltransferase (FUT3), respectively. These enzymes play a role in the production of fucosylated HMOs [26]. In the case of women whose FUT2 and FUT3 enzymes have been deactivated, the quantity of α1–2-fucosylated HMOs in their milk is either low or completely absent. As a result of mutations in both alleles of the Se gene, the activity of FUT2 can be rendered inactive, whilst the activity of FUT3 can be decreased. Both of these mutations can occur simultaneously. The milk of Lewis-negative mothers contains no α1–4-fucosylated HMOs or only very small amounts. Mothers who possess the Se gene produce the FUT2 enzyme, whereas those who do not possess the gene do not produce FUT2. The interaction between FUT2 and FUT3 enzymes results in the formation of two primary Lewis’s antigens, Lea and Leb. This interaction also leads to the development of four common milk phenotypes: Se+/Le^(a−b+)^, Se-/Le^(a+b−)^, Se+/Le^(a−b−)^, and Se−/Le^(a−b−)^ [27,28].

**Figure 2:**
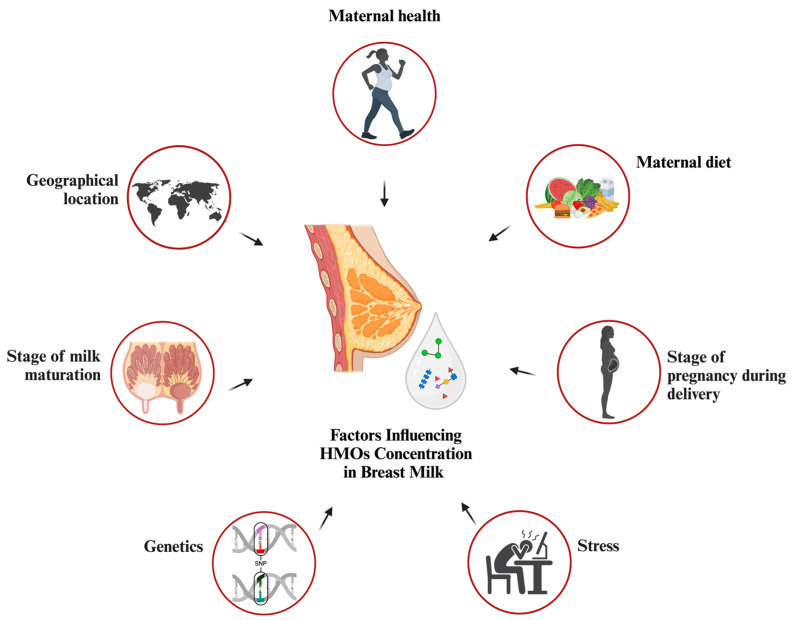
Factors influencing HMOs concentration in breast milk [31].

Colostrum is known to have high concentrations of HMOs, but variables other than the duration of lactation that contribute to the variation among women have not been investigated. There are data indicating that there is variation in the prevalence of HMOs among different populations [29]. This variation may be largely attributed to the uneven distribution of milk groups in different geographical regions [30]. Potential non-genetic factors that could influence the difference in HMO contents include the age of the mother, her dietary habits, thereby affecting their body-mass index (BMI), and any severe health conditions she may have (Figure 2) [31].

According to the findings of the research carried out by McGuire *et al.*, the concentration of 3′-Fucosyllactose (3′-FL) was at least four times higher in milk collected in Sweden compared to milk obtained in rural Gambia among healthy nursing women of eleven different nationalities. On the other hand, the concentration of disialyllacto-*N*-tetraose (DSLNT) was nearly four times lower in Sweden compared to the concentration in rural Gambia [32]. Moreover, in Gambia, lactating moms generate far fewer LNnT during the wet season compared to the dry [33]. After a cesarean section, the quantities of 3′-SL, 2′-FL, and 6′-galactosyllactose (6′-GL) in human milk were found to be lower than those seen in vaginal birth, according to the findings [34]. In addition, parity also impacts the concentration of HMOs. The impact of parity on HMO content is probably influenced by the association between maternal BMI and the composition of fatty acids in human milk, as well as the concentration of fat and protein. This correlation tends to rise with each subsequent live birth [35]. Concerning the impact of prematurity on HMOs, maternal milk showed elevated levels of 3′-SL, 6′-SL, LNT, and LNDFH-I following preterm delivery compared to term delivery. Furthermore, the ratios of 3′-SL and 6′-SL varied significantly depending on the stage of milk maturation [36,37].

Furthermore, apart from the natural factors genetic secretor status and lactation duration), variations in reported HMO concentration are also associated with different inter-laboratory approaches for glycan analysis. Therefore, the current collection of work on HMO quantification is a valuable tool for determining the average concentrations of certain HMO structures and studying their changes during breastfeeding [38]. The findings indicate that the concentration of HMO is constantly changing during lactation, with around 15 distinct HMO structures making up the bulk (>75%) of the overall HMO fraction. Based on the descriptive analysis of the current literature. More precisely, the main components of the total HMO composition in mature milk are 2′-FL, LNDFH-I, LNFP-I, LNFP-II, LNT, 3′-FL, 6′-SL, DSLNT, LNnT, DFL, FD-LNH, LNFP-III, 3′-SL, LST c, and TF-LNH. The smaller HMOs, namely, hexasaccharides, dominate in terms of relative molar abundance ratio [39].

The amount of HMOs and the different types of HMOs that are found in breast milk are the subject of a great number of research studies. During the first 14 weeks of breastfeeding, the concentration of HMO was found to be 9 g/L, according to the findings of an experiment that utilized reversed-phase high-performance liquid chromatography. By the time one year after giving birth, it had progressively declined to 4 g/L [40]. In another investigation, high-performance anion exchange chromatography linked to pulsed amperometric detection (HPAEC-PAD) was employed to uncover the peak concentration of OSs on day 4 postpartum (20 g/L), which subsequently declines by around 20% by day 30 of lactation [41]. Therefore, the same strategy was employed to investigate HMO concentration using high-pressure liquid chromatography. Researchers also analyzed correlations between the composition of HMOs and infant growth and development. Based on the research findings, greater diversity and evenness of HMOs at 1 month were linked to reduced total and percentage fat mass. Elevations in LNFP I levels after 1 month were linked to a decrease of 0.40 kg in premature weight, 1.11 kg in body weight, and 0.85 g in lean mass. Following 6 months, LNFPI was linked to a 0.79 g decrease in fat mass, whereas disialyl-LNT and LNFPII resulted in 1.92 g and 0.42 g increases in fat mass, respectively. Elevations in fucosyl-disialyl-lacto-*N*-hexaose and LNnT levels were linked to a 0.04% increase in body fat and a 0.03% decrease in body fat, respectively [42]. More specifically, 3′-SL and 6′-SL found in human milk were separated and investigated using graphitized carbon liquid chromatography–mass spectrometry (LC-MS) by Csernák *et al.* [43] During the postpartum period, milk samples were collected and analyzed. Within the study period, the concentrations of 3′-SL remained mostly consistent in colostral milk, ranging from 205.8 to 252.4 µg/mL during the first lactation period and from 134.9 to 208.8 µg/mL during the second one. In mature milk, the concentration was also relatively constant, ranging from 224.6 to 248 µg/mL and from 165.3 to 180.6 µg/mL, respectively. The concentrations of 6′-SL (53.1–939.1 µg/mL), which is the primary acidic HMO in this work, were higher than the quantities reported by previous studies [44,45]. Although the concentrations of 6′-SL did not decrease considerably during the first week of lactation, a declining tendency started between the 7th and 30th day and persisted until the end of the study (6 months), consistent with the findings of Thurl *et al.* [46].

Considering HMOs’ concentration in milk, colostrum (0–5 days) had the greatest total HMO concentration, measuring 17.6 g/L, which then decreased consistently. Nevertheless, the quantity of breast milk consumed by the newborn grows following colostrum, during the transitional and mature phases. The concurrent rise in milk consumption and reduction in HMO concentrations is anticipated to counterbalance the quantity of HMO ingested by the newborn [47].

As a result, human milk includes around 200 distinct HMO structures, with a significant proportion of these structures being present in low quantities in comparison to abundant structures. Throughout the breastfeeding process, individual HMOs are both varied and dynamic. Analysis of lactation phases in the study reveals that total HMOs are higher during the early stages of lactation and decrease after the colostrum period. Although certain exceptions occur where specific structures fluctuate, remain stable, or increase during the post-colostrum phase, most of the individual structures seem to diminish consistently throughout breastfeeding. After colostrum, 3′-FL levels rise dramatically, as documented by Borewicz *et al.* (2020) [48], Wang *et al.* (2020) [49], and more recently by Plows *et al.* (2021) and Gu *et al.* (2021) [50]. A study by Plows *et al.* (2021) found that 3′-FL grew tenfold from 1 month to 24 months [51].

Recent developments have resulted in the introduction of an infant formula that contains five HMOs, namely, 2′-FL, LNnT, LNT, DFL, 3′-SL, and 6′-SL HMOs. Knowing the most representative composition of human milk based on healthy mothers all over the world is essential in order to achieve the continuous objective of designing infant formulas that closely mimic human milk in terms of both their composition and their functionality. This information can help in enhancing the formulations. Due to the need of obtaining estimated numbers that accurately represent the entire population, the authors opted for a combined overview when including HMOs in infant formulas and other nutritional compositions [47].

In conclusion, there is a major impact on newborn health brought about by the structural variety that is exhibited by HMOs as a result of genetic, environmental, and physiological variables. HMOs have the ability to selectively promote healthy gut bacteria, such as *B. infantis*, which are able to make effective use of fucosylated and sialylated health maintenance organisms. The presence of non-secretor mothers who have lower levels of fucosylated HMOs may result in the development of children with altered gut microbiota composition. This is because the growth of some bacterial strains is inhibited. Through their ability to prevent pathogens from adhering to the gut epithelium, HMOs have the ability to mold the microbiome and affect the immune system. Because of reduced levels of protective HMO, infants with mothers who are not secretors may be at a greater risk of contracting infections. In addition to their anti-inflammatory properties, sialylated HMOs are beneficial to the development of the immune system. Sialylated HMOs are essential for the development of cognitive abilities because they serve as precursors for gangliosides and glycoproteins in the brain. Differences in HMO levels can have an impact on the availability of these substances during developmental stages. The presence of early HMO diversity can have an effect on the metabolism, intestinal health, and immunity, which may in turn influence the likelihood of developing chronic illnesses later in life.

## 3. Synthesis and Metabolism

### 3.1. Synthesis

During the biosynthetic process, the polymorphism of several genes contributes to the substantial variability of HMOs with regard to their composition [52]. It is believed that glycosyltransferases in the mammary gland are responsible for catalyzing the formation of the vast majority, if not all, of HMOs. These HMOs are generated from Lac at the non-reducing ends [53]. The secretory vesicle is most likely where Lac and other OSs concentrate before being exocytotically fused with the apical plasma membrane and released [54]. In the mammary gland, β1–4-galactosyltransferase 1 (β1–4GalT1) forms a lactose synthase complex with α-lactalbumin to synthesize Lac itself [55]. Nevertheless, the majority of the particular glycosyltransferases that are in charge of creating HMOS structures with certain glycosidic connections have not yet been found. Human FUT2, which is encoded by the Se gene, and FUT3, which is encoded by the Le gene, are the best-understood examples. These enzymes are in charge of forming α1–2-linked fucosides in human mammary glands, respectively [56]. The FUT2, which is expressed when the Se gene is activated, is in charge of Fuc extending the terminal Gal of the type-1 chain of HMOs via α1–2 linkage [57,58]. The Le gene facilitates the development of FUT3, which attaches Fuc with α1–3/4 linkage to a subterminal GlcNAc of the type-1 chain of HMOs [59,60]. Mothers can be categorized into four distinct categories based on the varying expressions of the Se gene and Le gene, classed as either positive (+) or negative (−) for both genes. Se(+)Le(+), Se(−)Le(+), Se(+)Le(−), and Se(−)Le(−) include 70% Se(+)Le(+) [58], 20% Se(−)Le(+), 9% Se(+)Le(−), and 1% Se(−)Le(−) [32,61].

Furthermore, the processing of core OSs enhances the variety of HMO structures in human milk. This course activates four glycosyltransferases: β3-galactosyltransferases and β4-galactosyltransferases facilitate Gal relocation, whereas β1,3-*N*-acetylglucosaminyltransferase (iGnT) and β1,6-*N*-acetylglucosaminyltransferase (IGnT) are implicated in GlcNAc transfer [62,63].

In addition to the many genes involved in HMO biosynthesis, it is hypothesized that additional factors also impact the endogenous production of HMO. Although the overall concentration of HMOs diminished considerably throughout breastfeeding, a more pronounced reduction in HMOs might result from seasonal variations and specific maternal dietary circumstances [64]. In Gambia, breastfeeding mothers who breastfeed during the rainy season produce milk with reduced HMO concentrations compared to those nursing in the dry season, when food availability is greater and energy intake is up [33].

### 3.2. Metabolism

Upon ingestion by the breast-fed newborn, HMOs exhibit resistance to both the low stomach pH and breakdown by the infant’s pancreatic and brush border enzymes [65,66]. Roughly 1% of the consumed HMOs are absorbed, enter the infant’s systemic circulation, and are eliminated unaltered in the infant’s urine [67,68]. Most HMOs are either broken down by the infant’s gut microbiota or eliminated unaltered with the infant’s stool [69]. Because HMOs are absorbed and transported throughout the body, they may have an effect on organs other than the stomach, such as the liver, brain, respiratory system, and urinary tract, suggesting that their biological activities may not be limited to the gut [8]. Glycoside hydrolases (GHs) and/or membrane transporters are necessary for the full breakdown of HMOs with various molecular configurations. The newborn gut microbiota has these enzymes and protein transporters, which are necessary for the gut bacteria to absorb, metabolize, and use HMOs. Thus, knowledge of the genes encoding the necessary transporters and enzymes can aid in identifying the methods and approaches for HMO consumption and breakdown. Numerous bifidobacterial species that are among the most active for the metabolism of HMOs in the gut microbiota of infants have had several of these genes discovered and identified [13]. Additionally, specific bacteria that can catabolize HMOs will become more prevalent than others based on the amount of HMOs consumed [70]. These bacteria act as probiotics, influencing the intestinal microbiota of the newborn [71,72]. Furthermore, several *Bacteroides* and *Lactobacillus* species, including *B. fragilis*, *B. vulgatus*, and *Lactobacillus casei* (*L. casei*), have demonstrated a high HMO metabolic ability [73].

#### 3.2.1. HMO Enzymatic Degradation by Glycoside Hydrolase Enzymes

In the presence of bacteria that possess the enzymatic capabilities necessary to degrade HMOs, they serve as an excellent substrate. As a result, it is believed that this will stimulate the microbial community to support the dominance of bifidobacteria in the gut microbiome throughout the early stages of life. In order to clarify the whole breakdown pattern of HMOs, research over the past ten years has mostly concentrated on characterizing the enzymes of bifidobacterial (*Bifidobacterium breve* (*B. breve*), *Bifidobacterium longum* (*B. longum*)) organisms. The enzymes that break down the typical prebiotics, galacto-oligosaccharides (GOS) and fructo-oligosaccharides (FOS), which are introduced to infant formulas, have also received the appropriate attention [74]. GHs, a complex repertoire of enzymes necessary for the structure-specific breakdown of HMOs, cleave glycosidic linkages between distinct HMO structures to liberate component monosaccharides or disaccharides as a precursor to further metabolism [75].

**Figure 3:**
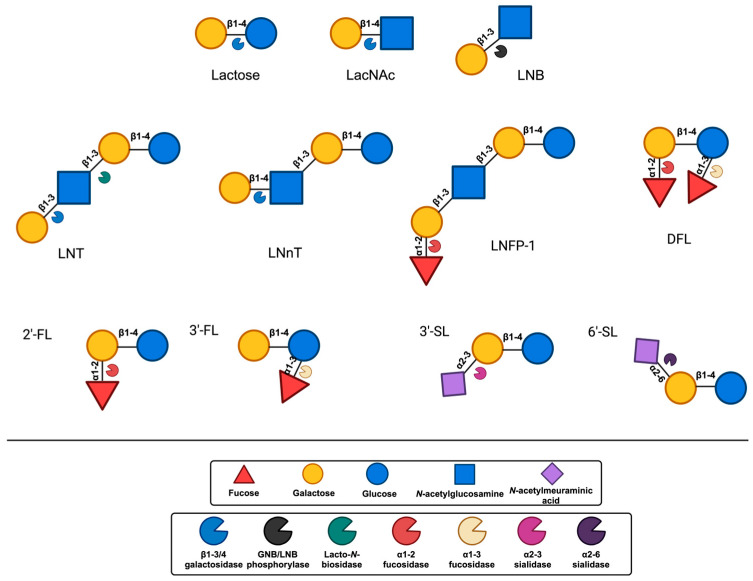
Types of Glycoside Hydrolases and Their Activities on HMOs [19,80].

The metabolism of HMO structures, including sialylated, neutral, and neutral fucosylated HMOs, depends on GH enzymes such as lacto-*N*-biosidases (LnbX), β-galactosidases, β-galactosidases, and fucosidases. Sialidases, conversely, are enzymes that facilitate the release of Neu5Ac from the core structure by targeting the α-2,3 and α-2,6 links [76]. Fuc and Neu5AC released from the core structures can be further metabolized by bacteria [77]. These gut microbiome-associated enzymes, which can be found extracellularly or intracellularly, are crucial for infant-associated gut microbes. Degradation of the HMO structure can be shown by comprehending the actions of these enzymes [22]. *β*-*N*-acetylglucosaminidases further break down the HMO complexes by cleaving the link between GlcNAc and Gal. Numerous enzymes of this kind have been documented in the literature; each has a predilection for the particular HMOs that are targeted, and some can work on both β-1,3 and β-1,6 links equally, while others favor a certain linkage type (Figure 3) [78,79].

Endo-*β*-*N*-acetylglucosaminidases from the GH18 or GH85 families are required to liberate OSs when glycans are conjugated to peptides as glycoproteins [19,80]. GlcNAc residues, also known as Sia residues, are cleaved by 2,3-2,6-a-sialidases from the GH33 family. Depending on their specificity, α-L-fucosidases, which are part of GH29 and GH95, eliminate decorated Fuc from the milk of Secretor mothers as well. In the major HMO chain, LNB/GNB phosphorylases of the GH112 family catalyze the hydrolysis of the β1–3 bond in LNB and the β1–4 bond in LacNAc. The GH20 and GH136 families’ LnbX or β-hexosaminidases/β-1,6-*N*-acetylglucosaminidases are responsible for releasing Lac from the nearby GlcNAc. β-galactosidases that can hydrolyze β1–4 links target the residual Lac from HMOs and liberate human milk Lac. A different and more condensed GH profile is needed for the breakdown of typical infant formula OSs, GOS, and FOS. The breakdown of GOS is dependent on the previously described GH2 and GH42 family enzymes, and their structure is less complicated. Enzymes from the GH13, GH32, and GH68 families that cleave the Fuc moieties from the OSs are mostly needed for the use of FOS [74].

#### 3.2.2. Species-Level HMO Metabolism

##### HMO Utilization Strategies of *Bifidobacteria*

Bifidobacteria are the predominant bacteria in breast-fed newborns, adept at metabolizing prebiotic components associated with human milk, especially HMOs, with certain species being notably widespread and numerous in the feces of healthy infants [81]. The carbohydrate metabolic capacities vary considerably across *Bifidobacterium* species, with *B. infantis* and *B. bifidum* being characterized as enthusiastic consumers of HMOs, due to their genetically encoded capacity to synthesize GHs enzymes necessary for the catabolism of various HMO linkages. Cross-feeding, a microbe–microbe interaction in which carbohydrate components generated by intestinal bacteria are made accessible as substrates for other members of the gut microbiome, limits *B. breve* and *B. longum* to less complicated HMOs or HMO-specific components. The gut microbiota benefits from increased interactions and the creation of beneficial compounds [82].

About 13.7% of the genomic content of bifidobacteria is devoted to the metabolism of carbohydrates. As was previously established, bifidobacteria have two primary ways to break down HMO structures: intracellular and extracellular degradation [81]. HMO degradation is a species-specific capacity, and strains of *B. infantis* and *B. breve* are frequently linked to intracellular degradation, in which complete HMOs are imported into the cell and metabolized into their monosaccharide components using OS transporters. This is very different from the extracellular degradation mechanism that *B. bifidum* strains most frequently exhibit [83]. The HMO degradation strategy employed by *B. longum* strains relies on the presence or absence of extracellular fucosidase and sialidase enzymes, as well as the lnbX gene encoding the LnbX. Strains possessing the lnbX gene utilize extracellular glycosidases, whereas those lacking lnbX necessitate OS transporters to internalize HMOs [84].

Three distinct mechanisms for host-glycan consumption have been identified in *bifidobacteria. B. longum* subsp. *infantis* encodes ATP-binding cassette (ABC) transporters for the internalization of intact OSs, which are then destroyed by intracellular GHs [85]. Certain strains of *B. breve* employ a comparable mechanism [86]. Conversely, *B. bifidum* secretes several GHs and assimilates the resultant monosaccharide or disaccharide residues [83]. A third method is employed by “scavenger” bifidobacteria, including *B. breve* and *B. longum* subsp. *longum*, which can consume only a limited portion of HMOs, occasionally relying on other species, such as *B. bifidum*, that can perform extracellular hydrolysis of larger HMOs [87].

*B. breve* is a common species in the intestines of infants, with various strains metabolizing HMOs according to their genetic composition. Only bacteria encoding GH29 α-fucosidase can use fucosylated HMOs, whereas GH95 α-fucosidase is encoded by *B. breve*. Certain strains exhibit a significant ability for the degradation of sialylated HMOs, with *B. breve* SC95 demonstrating a preference for sialylated HMOs. An external β-galactosidase releases Gal and lacto-*N*-triose II, which are then the substrate of a *β*-*N*-acetylglucosaminidase that creates GlcNAc and Lac, metabolizing LNnT extracellularly in *B. bifidum*. In a separate research, β-galactosidase BbgIII was shown to be an external membrane-anchored multidomain glycosidase that belongs to the GH2 family out of the five β-galactosidases expressed by *B. bifidum* JCM1254. BbgIII is unable to hydrolyze fucosylated derivatives of LNnT and LacNAc and preferentially operates on the β-1,4 bond of these glycans [88]. Although it employs a different β-galactosidase, *B. infantis* follows a similar approach for LNnT degradation as for LNT. The HMO gene cluster contains the encoding gene for Bga2A, a GH2 β-galactosidase that internalizes LNnT and hydrolyzes it into Gal and lacto-*N*-triose II. Although it does not hydrolyze LNT, this enzyme is particularly active on Lac and also effectively breaks down LacNAc and LNnT [89].

*B. infantis* is frequently regarded as the primary species that utilizes HMOs in the infant gut. Numerous studies demonstrate that *B. infantis* strains can significantly proliferate on various specific HMOs, including 2′-FL, 3′-FL, LNT, LNnT, 3′-Sialyllactose (3′-SL), 6′-Sialyllactose (6′-SL), LNFP-I, and LDFT (lactodifucotetraose) as the sole carbon source. These strains exhibit superior HMO catabolic capabilities compared to other bifidobacterial species, such as *B. longum* subsp. *longum*, *B. breve*, and *Bifidobacterium adolescentis* isolates. This trait is consistently preserved across the subspecies [90]. A 43 kb gene cluster unique to HMO breakdown is present in *B. infantis* strains. All of the GH enzymes needed to effectively cleave HMOs are encoded by this gene cluster, including the GH95 family’s 1,2-α-fucosidases, the GH29 family’s 1,3/4-α-fucosidases, the GH33 family’s 2,3/6 sialidases, the GH20 family’s *β*-*N*-acetylhexosaminidase enzymes, the GH2 family’s β-galactosidases, and the GH42 family’s LNT β-galactosidases [91]. Strains of *B. infantis* utilized a range of HMOs intracellularly, including LNT, LNnT, LNB, fucosylated HMOs, and sialylated HMOs. These complexes are absorbed and degraded by exoglycosidases in the cytoplasm for monosaccharide metabolism [92]. The ability to utilize *N*-glycans from glycoproteins is correlated with the presence of genes producing endo-*β*-*N*-acetylglucosaminidases (from the GH18 and GH85 families), according to genome analysis in *B. longum* subsp. *longum*, *B. longum* subsp. *infantis*, and *B. breve*. The enzymes EndoBI-1 (GH18) from *B. infantis* ATCC 15697, EndoBI-2 (GH18) from *B. infantis* SC142, and EndoBB (GH85) from *B. longum* DJO10A have been described [93,94]. They possess one or two transmembrane helices and are capable of deglycosylating extensively glycosylated bovine RNaseB. EndoBI-1 and EndoBI-2 further deglycosylate human and bovine lactoferrin. EndoBI-1, an enzyme that is constitutively produced and heat-stable (95 °C for 5 min), has been demonstrated to break the glycosidic bond of ChbNAc in proteins with rich mannose or other complex *N*-glycans [95,96,97,98].

The presence of particular genomic clusters linked to HMO utilization has been discovered through genome sequencing of *Bifidobacterium pseudocatenulatum* (*B. pseudocatenulatum*), a commonly found infant gut-associated species with a number of purported health benefits, such as the capacity to bind mutagenic aromatic amines and lower cholesterol levels [99,100]. Recent research has focused on determining whether or not *B. pseudocatenulatum* is able to survive just on 2% pooled HMOs as its sole supply of carbohydrates. The growth of various strains of *B. pseudocatenulatum* that originated from neonates was demonstrated to be successful in HMOs. Among these strains, some of them consistently used LNT and LNnT. In addition, the findings of the study demonstrated that the utilization of fucosylated HMO was influenced by the presence or absence of genes that encode specific α-fucosidases belonging to the GH29 and GH95 families [101].

##### HMO Utilization Strategies of *Bacteroides*

*Bacteroides* are prevalent components of the gut microbiota, recognized for their capacity to metabolize various OSs, polysaccharides, and host-derived glycans, including mucus [102]. It has been demonstrated that members of the genus *Bacteroides* use milk glycans, including HMOs, as fermentable sources of carbohydrates to develop. Certain gene clusters known as polysaccharide utilization loci (PULs), which are involved in mucin consumption, exhibit increased expression when cultivated on HMOs. On a regular basis, PULs are responsible for the encoding of sensor regulators, GHs, and homologs of two outer membrane proteins known as SusC and SusD. These proteins assist in the binding and importation of starch. Each PUL within a *Bacteroides* genome seems to be tasked with detecting and using a certain kind of OS or polysaccharide. PULs are likely to participate in the utilization of HMO [102]. It is possible that *Bacteroides* can break down HMOs by employing parallel metabolic processes since mucins and HMOs have structural similarities. Prior to an initial surface hydrolysis, *Bacteroides* bind complex OSs, including mucin glycans, on the cell surface. This allows the resultant OS to pass past the outer membrane of SusC-like porins and into the periplasm for further degradation [103].

By preventing direct contact between gut bacteria and the gut epithelium and permitting the transport of small molecules from the lumen to the epithelium, mucin glycoproteins contribute significantly to the protection of the gut epithelium. Gut bacteria including *Bacteroides* and *Akkermansia* can break down the mucin glycocalyx, whereas other bacteria, such as *B. infantis*, cannot consume mucin glycans. From this background, Karav *et al.* evaluated the effects of several gut microbiomes on colonic mucin breakdown by comparing the untargeted MS profiles. Samples from nine newborns colonized by *B. infantis* EVC001 were compared with samples from ten children colonized by elevated amounts of mucolytic taxa, including *Bacteroides*, serving as controls. The research indicates that infants colonized by *B. infantis* have diminished mucin breakdown, characterized by a reduction in both the number and variety of liberated colonic mucin-derived *O*-glycans, with a negative connection with Bifidobacteriaceae. *B. infantis* is unable to break these *O*-glycans, resulting in diminished Bacteroidaceae populations [104].

Both *B. fragilis* and *B. vulgatus* have been shown to be susceptible to HMO treatment. The utilization of MALDI-FTICR MS for the examination of HMO consumption reveals that, in contrast to the predilection for short-chain glycans that is exhibited by *Bifidobacterium* species, *Bacteroides* makes effective use of a wide variety of HMO glycans [71]. The superior use of HMO by *B. fragilis* in comparison to *B. vulgatus* indicates that certain *Bacteroides* species may be more adept at exploiting HMO during the colonization of the newborn gut [105]. Kijdel *et al.* investigated the HMO consumption ability of several *Bacteroides* isolates by conducting growth curve analyses on six prevalent HMOs (2′-FL, DFL, 3′-SL, 6′-SL, LNT, LNnT) following the isolation of *Bacteroides* bacteria from newborn feces samples using an optimized technique. Isolates frequently exhibited analogous growth characteristics on structurally comparable HMOs, such as sialylated or fucosylated sugars. Researchers observed variability in HMO usage across different strains of the same species and decided to concentrate on a *Bacteroides dorei* (*B. dorei*) isolate capable of using the test HMOs. Unexpectedly, there was no evident up-regulation for the majority of GH families previously identified as degrading HMOs, perhaps due to their predominant characterization in *Bifidobacterium* species. *B. dorei* has a broad response to HMOs, significantly up-regulating many shared GH families under all circumstances [106]. The type strains *B. fragilis* (Bfra_TS), *B. vulgatus* (Bvul_TS), and *Bacteroides thetaiotaomicron* (*B. thetaiotaomicron*) (Bthe_TS)) have demonstrated robust growth on 1% 2′-FL, 3′-FL, and DFL as the exclusive carbohydrates available [107].

According to the findings, *Bacteroides* species are able to hydrolyze HMOs in an opportunistic manner because of their structural similarities to mucin. On the other hand, *bifidobacteria* have produced genes that allow them to selectively use glycan structures that are specific to human milk. The evidence presented here throws clarification on the reason why bifidobacteria may be found in the infant gut in larger relative abundances than other bacteria that use HMO.

##### HMO Utilization Strategies of *Lactobacillus*

Members of the genus *Lactobacillus* appear to have a limited capacity to use this carbon source, and their usage of HMOs is a comparatively unexplored area in comparison to bifidobacteria. Intestinal lactobacilli often have a wide variety of glycosyl hydrolases in their genomes [108].

Although the majority of the information on the use of HMOs pertains to *Bifidobacterium* species, it has recently been revealed that some species of *Lactobacillus* are capable of metabolizing certain HMOs in order to maintain their development [107,109]. Lactobacilli that are found in the digestive tract often have genomes that contain a wide variety of GHs [110], likely facilitating the utilization of dietary carbohydrates. Sugar transporters, with the exception of GlcNAc, are involved in the transportation of mono- and disaccharides, which are not primarily connected with host glycans. This is the case for a large number of sugar transporters that have been found. The phosphoenolpyruvate-dependent sugar phosphotransferase systems (PTS) that are present in lactobacilli are responsible for the internalization of this monosaccharide. These systems are predominantly found in the mucosae of the host, and the genes that are connected with them are activated in the intestinal tract of animal models [111].

Most of the knowledge on lactobacilli’s metabolism of HMO is restricted to strains of the *Lacticaseibacillus casei-paracasei-rhamnosus* group. In lactobacilli, three α-L-fucosidase enzymes have been identified, albeit only in a few species. For this species, sialidase enzymes for sialylated HMOs have not been identified. The action of *L. casei’s* α-L-fucosidases on brief, FucOS suggests intracellular hydrolysis [112,113].

Few investigations have been undertaken about the metabolism of HMOs by *Lacticaseibacillus rhamnosus*, previously referred to as *Lactobacillus rhamnosus*. Whole genome sequencing of *L. rhamnosus* GG discovered genes that encode α-L-fucosidases, whereas sequencing of *L. rhamnosus* HN001 uncovered genes producing α-L-fucosidases [113]. The occurrence of the latter is uncommon in *L. rhamnosus* and in lactobacilli overall, with *L. casei* being the sole other *Lactobacillus* species identified thus far that encodes α-L-fucosidases capable of hydrolyzing fucosylated HMOs *in vitro* [112].

Although certain strains of *Lactobacillus acidophilus* (*L. acidophilus*) have genes that produce enzymes for hydrolyzing specific HMOs, their ability to thrive on HMOs is frequently restricted and highly strain dependent. *L. acidophilus* NCFM is a particular strain that exhibits notable growth on various HMOs [71]. *L. acidophilus* NCFM has been demonstrated to use the neutral, type II HMO LNnT, exhibiting growth between 0.6 and 1.0 as determined by OD600nm on 1% LNnT as the only carbohydrate source [109]. Wang *et al.* investigated the capacity of *L. acidophilus* to proliferate on certain carbohydrate substrates, such as Glc, HMOs, GOS, XOS, and 2′-FL. After 48 h, *L. acidophilus* NRRL B-4495 absorbed HMOs and modestly ingested HMO 2′-FL. The strains LDFT, LNT, LNFP, and LNDFH used HMOs, generating organic acids like as lactic acid and butyric acid, along with minor quantities of propionic and valeric acid [114]. A “scavenger” strategy may be utilized by lactobacilli, *B. breve* and *B. longum*, in order to utilize short glycans acquired from the host that are created by other members of the gut microbiota [115].

#### 3.2.3. HMO_S_ Metabolism By-Products

The interaction between microbial metabolites and the host is one of the processes by which the gut microbiota shapes health. The intestinal fermentation of dietary indigestible carbohydrates produces some of these potentially advantageous metabolites, including HMOs in the neonatal population. Among the most researched microbial metabolites are SCFAs, with acetate, butyrate, and propionate receiving special attention. SCFAs can affect host physiology and the immune system in addition to serving as a source of energy for colonocytes [116,117].

The variation in intestinal SCFA composition throughout the first few months of life most likely reflects changes in the gut bacteria community. Although most intestinal bacteria make acetate, the most prevalent SCFA in the gut, only a small portion of gut colonizers can manufacture butyrate and propionate [118]. Due to these differences, acetate is present in detectable levels from birth, but the concentrations of propionate and butyrate increase throughout the first year of life as the bacteria that make them colonize the body [119]. Rather, lactate, succinate, and SCFA formate—other less well-known SCFA-associated compounds—were more common in the first few months of life and decreased until the age of one year [120].

Butyrate is a necessary SCFA metabolite that is produced in the human colon by HMOs through cross-feeding with bacteria that produce it [121]. Histone deacetylases are inhibited, and G protein-coupled receptors are activated by butyrate, a local energy source for gut colonocytes that has a variety of physiological consequences [122]. On the other hand, both the intestinal lumen and the peripheral circulation contain acetate, which can influence hunger through a central homeostatic mechanism and drive fat formation via the GPR43 signaling pathway [123]. Propionate is transported to the liver, where it performs a number of physiological processes and serves as a substrate for gluconeogenesis [124]. However, the effects of short-chain hydroxyl-fatty acids such as lactate, and other metabolites generated from the gut microbiota (such as formate and succinate) are still mostly unknown [118,125].

The relationship between HMOs, *B. infantis* supplementation, and SCFA production is supported by research by Frese *et al.* (2017). When compared to breast-fed babies, the amounts of lactate, acetate, butyrate, and formate found in the feces of infants who were supplemented with a commercial strain of *B. infantis* were shown to be significantly reduced [126]. Using an anaerobic culture, the researchers investigated the effect that primary fucosylated and sialylated HMOs had on the growth and metabolic output of a variety of microbiota bacteria. They discovered that *Bifidobacteria* and *Bacteroides* species flourished when they were given 2′-FL, 3′-FL, and LDFT. On the other hand, *Lactobacillus delbrueckii*, *Enterococcus faecalis*, and *Streptococcus thermophilus* exhibited a lack of growth and a reduction in pH. Numerous strains of *B. longum*, *B. vulgatus*, and *B. thetaiotaomicron* exhibited elevated levels of neuraminidase activity and generated substantial quantities of lactate or SCFAs [127]. Based on the results of *in vitro* experiments conducted on human microbiota that have been cultivated, it has been proven that two particular HMOs, namely, 2′-FL and LNnT, had the ability to induce the synthesis of *Bifidobacterium* and SCFA [128].

#### 3.2.4. Advanced Analytical Methods for HMO Characterization

Comprehensive analytical techniques for the separation of OSs from human milk samples are still being developed, despite the fact that the study of glycan structures constitutes a distinct scientific discipline, i.e., a subfield of glycomics. In order to discover the biological purpose of these oligosaccharide structures and, as a result, design infant formulas that are more suited, it is of the utmost importance to elucidate these structures. Because of their several isomers, HMOs are complex structures with varying monosaccharide building block positions and connections that necessitate sophisticated techniques for in-depth investigation [129]. The characterization of milk OSs has traditionally been performed using nuclear magnetic resonance (NMR), high-pH anion exchange chromatography with pulsed amperometric detection (PAD), or lectin affinity. Large numbers of additional analytical techniques have been applied since the introduction of MS for the analysis of oligosaccharides. These techniques include hydrophilic interaction chromatography and porous graphitic carbon (PGC) separations, either with or without coupling to MS or stand-alone matrix-assisted laser desorption ionization (MALDI) MS [130].

LC and capillary electrophoresis (CE) are the liquid phase separation techniques that are utilized for HMO analysis the most frequently. LC employs several stationary phases that provide reversed phase, anion exchange, porous graphitized carbon, and hydrophilic interaction modes. The primary limitation of LC methods is the necessity for oligosaccharide standards to ascertain retention time for structural elucidation in conjunction with publicly accessible glycan libraries. However, these standards are often commercially unavailable, and when they are, they tend to be prohibitively expensive or of insufficient purity [131]. For CE, derivatization of mostly uncharged carbohydrates is essential to facilitate their appropriate electromigration. The use of a charged fluorescent marker, such as 9-aminopyrene-1,3,6-trisulfonic acid (APTS), enhances the method’s sensitivity to the 10^−11^–10^−12^ M range [132]. In 2007, Bao *et al.* designed and validated a capillary electrophoresis-based technique for the measurement of 12 principal acidic HMOs utilizing UV detection [133]. In order to determine the presence of HMO standards and OSs in human milk samples, Volpi and his colleagues devised a CE technique that utilized simple UV detection at a wavelength of 254 nm. In spite of the presence of Lac and other high-concentration contaminants, such as an excess of fluorophores, proteins, and salts, this CE method was able to successfully separate the primary neutral and acidic OSs from breast milk [134].

HPAEC-PAD, which stands for high-performance anion-exchange chromatography with pulsed amperometric detection, has been utilized rather frequently for the purpose of separating HMOs due to its ability to resolve isomers [130]. Before proceeding, however, it is necessary to separate the neutral and acidic glycans. RP-HPLC, which stands for reversed-phase high-performance liquid chromatography, is yet another technology that is quite popular for HMO analysis [135]. On the other hand, hydrophilic interaction chromatography HPLC (HILIC) facilitates the separation of HMOs, enabling effective isomer differentiation, but requires oligosaccharide labeling with 2-aminobenzamide by reductive amination [136]. Micellar electrokinetic chromatography (MEKC) is proficient for naturally charged HMOs; nevertheless, existing methodologies are tailored for acidic HMOs and exhibit limited sensitivity [133]. In order to address the same sensitivity limits as MEKC, many capillary electrophoresis variations have been applied for HMO analysis. However, end-labeling is often required in order to accommodate these constraints [137]. As a result of the requirement for costly or non-existent standards, separation-based procedures, such as derivatization, have a primary drawback when it comes to comparing retention durations. This is especially true in HMOs, which is why mass identification is a vital analyzing tool [138].

In addition to being effectively employed for milk OS analysis for structural characterization, MS is widely used in the field of glycan analysis. The two most used soft ionization techniques for MS-based sugar analysis are electrospray ionization (ESI) and MALDI. In MALDI ionization, a laser beam is used to ionize the sample after it has been coated or mixed with an energy-absorbent matrix [139]. Using this technique, the analytes produce single protonated ions. In contrast, ESI uses an electric field to transform solution-phase analytes into gas-phase ions, producing multiple-charged ions [140]. In order to improve sensitivity and stabilize OSs during the ionization process, permethylation is a technique that is utilized. Additionally, permethylation produces detectable fragment ions and aids in preventing fucose rearrangement during MS analysis. The structural identification of HMOs is facilitated when MS is used in conjunction with chromatographic or electromigration separation techniques [141]. On the other hand, it necessitates additional processes and has the potential to make analysis more difficult due to incomplete derivatization. It is possible for standard RP columns like C_18_ to produce isomeric separation; however, they do not give thorough separation of isomeric species [142].

The offline MS profiling of HMO with MALDI TOF MS was initially reported by Stahl *et al.*, who successfully detected neutral oligosaccharides in positive mode as monosodium adducts, alongside acidic oligosaccharides in both positive and negative modes. De-sialylated fragments were found in the acidic fraction [143]. For example, an investigation of permethylated HMOs using LC-MS has been published by Oursel *et al.* [45], who also compare the analytical techniques with those of RPLC and PGC analyses. Through the utilization of a PGC column and an RPLC column, respectively, native HMOs and permethylated species were separated, and ion trap MS equipment was utilized to identify the separation. It was discovered that the PGC column was more effective for high-throughput species detection because of the lower amount of time required for sample preparation [144]. Identification and quantification of the free disaccharides LNB and LacNAc from human milk was accomplished by the development of a PGC stationary phase-based LC-MS/MS technique. The technique monitored the variations in concentration throughout the first week of breastfeeding and discovered that there were consistent declines. A better knowledge of HMO biosynthesis may be achieved by gaining a grasp of the ratio of free LNB to LacNAc in human milk. When the neutral and acidic HMOs were concurrently evaluated in a single run, the isomeric pair of LNT and LNnT was well separated. This was accomplished by the use of a sample preparation approach that was both rapid and straightforward. In this particular investigation, the lengthy chromatographic run time was a significant limitation [145]. All of the studies that were discussed concentrated their attention primarily on the low-molecular-weight oligosaccharides that were the most common, and they only reported a small number of HMOs. Within the context of a fairly recent and developing body of work, Stein and his colleagues developed a reference MS library of annotated HMOs that is searchable. The HILIC-ESI-MS/MS technique was used to describe the reference standards, and the library presently comprises 469 spectra of both positive and negative ions [146]. The library was able to accommodate the identification of unknown reduced or non-reduced oligosaccharides from human milk due to the fact that the reference standard combination comprised human milk from one hundred women who were nursing their children. Under circumstances in which standards are unavailable, this knowledge is a potentially fruitful alternative for future work. Another new technology that is being used in glycomics research is called CE-ESI-MS. It is necessary to combine the CE system with negative ESI detection in order to analyze negatively charged carbohydrates or sugars that have been tagged with APTS. When using this mode, the detection is very selective; nevertheless, it is less sensitive and more susceptible to the effects of corona discharge than when using the positive ESI mode. Multiple carbonyl-reactive target mass tag (TMT) tagged HMO standards were utilized in the development of a CE-ESI-CID-MS/MS technique, which was designed in order to alleviate the problem [147]

In summary, direct MS techniques may serve as a rapid option for HMO analysis because of the absence of separation requirements; nevertheless, they cannot differentiate isomeric structures, which may be essential for assessing if just particular types of links are impacted.

## 4. What Are Potential HMOs Functions?

**Figure 4:**
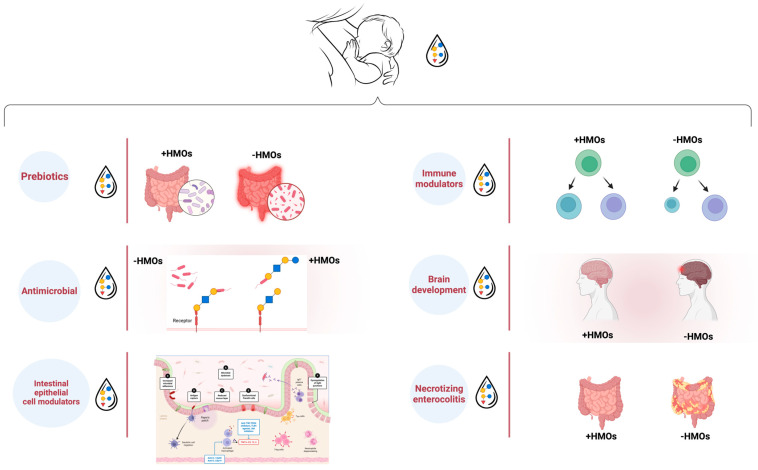
Beneficial effects of HMOs on human health [19].

Given the distinctive structure of HMOs, its impact on infant health and development has been extensively studied in the last ten years (Figure 4) [148,149,150].

### 4.1. HMOs as Growth Factors for Beneficial Bacteria

According to the definition provided by Roberfroid *et al.*, a prebiotic is a substance that has been fermented in a selected manner and enables particular changes to occur in the composition and/or activity of the microbiota in the gastrointestinal tract. These changes ultimately bestow advantages on the well-being and health of the host [151]. HMOs operate as metabolic substrates for particular bacteria such as *B. infantis.* Consequently, these bacteria exert a growth advantage and flourish. Other bacteria that are unable to avail themselves of HMOs have a drawback and exhibit reduced or no growth. Therefore, HMOs act as the initial prebiotics that people come across in their diet, often starting from the moment of their birth. The utilization of HMOs by bacteria necessitates a comprehensive array of enzymes, transporters, and complementary compounds. Some bacteria have developed alongside HMO enzymes and produce sialidases that break down Sia and fucosidases that break down Fuc. Just a few numbers of bacteria have the ability to break down the whole range of HMOs. Some bacteria can only selectively use a restricted range of HMOs or particular epitopes on more intricate HMOs [71,152]. For instance, bacteria possessing a specific fucosidase enzyme may be capable of taking up Fuc, but not Sia. Only after other bacteria have eliminated the Fuc or Sia from the backbone can some bacteria effectively use HMOs, resulting in a “community feast” where several bacteria can collectively break down the complete set of HMOs, but only when they function as a community [8].

**Figure 5:**
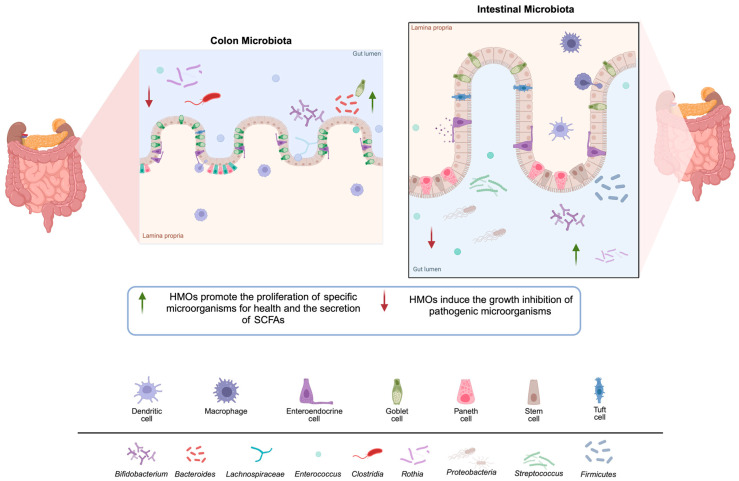
HMOs stimulate the release of SCFAs and the growth of particular gut bacterial strains that can be beneficial to the health of individuals [19,149].

Within the gut, HMOs have the ability to modify the microbiota, causing the growth of *Bifidobacterium*, *Firmicutes*, and *Actinobacteria.* In the colon, HMOs have the ability to induce the proliferation of *Bifidobacterium*, *Bacteroides*, and Lachnospiraceae. At the same time, HMOs reduce the number of bacteria that are detrimental to human health. These bacteria include *Enterococcus*, *Proteobacteria*, *Streptococcus*, and *Lactobacillus* in the gut, as well as *Rothia*, *Enterococcus*, and *Clostridium* in the colon area (Figure 5) [153].

The high concentration of HMOs in breast milk makes it one of the most potent deterministic variables for the selection of infant gut microbiota species. Generally regarded as helpful to the human body through a variety of processes, including the production of SCFAs, bifidobacteria are anaerobic Gram-positive bacteria that belong to the Actinobacteria phylum [154]. Through Tissier’s work at the Pasteur Institute, the concentration of bifidobacteria in the feces of newborns fed breast milk as opposed to formula was first observed in 1900 [79]. Important members of the *Bifidobacterium* genus selectively ingest these HMOs in the infant’s gut [155]. Between 50% and 90% of the entire bacterial population seen in breast-fed infants’ feces are bifidobacterial strains, making them the most prevalent colonizers in their guts [81]. In particular, *B. longum* subsp. *infantis* is an effective HMO-consuming species linked to infant health advantages.

Because of their extensive repertoire of carbohydrate-active enzymes, which enable certain bifidobacterial strains to utilize glycans generated from breast milk, such as HMOs, bifidobacteria have been successfully adapted to the newborn gut [153,156]. According to Gibson *et al.* (2017), HMOs function as prebiotics by encouraging the growth of good intestinal bacteria, which produce SCFAs that are essential for gut health. A prebiotic is “a substrate that is selectively utilized by host microorganisms conferring a health benefit” [157].

The precise mechanisms governing the seeding, expansion, and dominance of *Bifidobacterium* in the newborn gut remain inadequately understood; nonetheless, HMOs are regarded as a vital nutrient that facilitates the proliferation of HMO-catabolizing *Bifidobacterium* populations [158,159]. HMOs are intricate compounds that necessitate bacterial GHs and transport mechanisms for catabolism, rendering them a specialized nutrition accessible to only a select group of microbes. Research indicates correlations among HMO intake in breast-fed infants, the proliferation of specific *Bifidobacterium* strains, and elevated fecal acetate and lactate concentrations. The influence of human milk on the formation of the infant gut microbiota is ambiguous, attributed to the presence of bioactive components such as lysozymes, lactoferrin, and antimicrobial peptides [160,161,162,163,164].

Numerous studies have shown that high levels of HMO-consuming bifidobacteria were positively correlated with a significant decrease in fecal HMOs [165,166]. In order to catabolize HMOs *in vitro*, a particular β-galactosidase from *B. longum* was utilized. This enzyme was able to match the chemicals that were identified in newborn fecal samples [167]. The relationship between HMOs in breast milk and alterations in the makeup of the fecal microbiota in healthy newborns was investigated by Borewicz *et al.* (2019, 2020). They discovered that variations in the makeup of the fecal microbiota were linked to newborns’ capacity to utilize HMOs, especially 2′-FL. High levels of *Lactobacillus* and *Bifidobacterium* in infants were associated with greater rates of 2′-FL intake. *Bifidobacterium* abundances were greater in infants who consumed more lacto-*N*-hexaose [48,168]. *Bacteroides* are associated with the decomposition of sialylated HMOs referred to as 3′-SL, 6′-SL, LST a, LST b, and LST c. This research lends confirmation to the discovery made by Yu *et al.* (2013), which states that *B. fragilis*, *B. vulgatus*, and *B. thetaiotaomicron* utilize these as their only sources of carbon [127].

Two infants delivered vaginally had their feces sampled for research by De Leoz *et al.* (2015). While newborn B was nursed and supplemented with formula, newborn A was breast-fed from birth. 16S rRNA sequencing was used to investigate the microbial makeup. The findings indicated a change in the bacteria Bacteroidaceae and Bifidobacteriaceae from non-HMO-consuming to HMO-consuming. But in newborn A, *Bifidobacterium* species predominated, whereas in infant B, HMO levels sharply decreased [165].

Subsequent investigations examine the use of HMOs by *Bifidobacterium* spp. Ward *et al.* reported that *B. infantis* ATCC 15697 utilized HMOs as its exclusive carbon source [169]. Additionally, Garrido *et al.* demonstrated that isolates of *B. infantis* thrived on pooled HMOs and individual HMO sugars, whereas certain examined strains of *B. bifidum* were unable to grow using 2′-FL and 6′-SL as their sole carbon source [170]. Bifidobacteria predominantly metabolize HMOs, with some strains exhibiting enhanced fermentation capacities and increased uptake of particular varieties. Consequently, several research have endeavored to elucidate the processes by which bifidobacteria catabolize HMOs, demonstrating that the use of HMOs is consistently preserved among *B. infantis* strains, which ferment all types of HMOs [86,158]. By way of illustration, *B. infantis* ATCC 15697 is capable of metabolizing a wide variety of HMOs, including molecules that are sialylated and fucosylated [86]. GHs [110] and family 1 solute binding proteins (SBPs), which are a component of ABC transporters for HMOs [111], were up-regulated as a result of the expression of a large number of HMO-utilization genes found in a particular region of the genome, known as HMO cluster I [75].

Yu, Chen, Kling, *et al.* (2013) discovered that during the *in vitro* anaerobic fermentation, *Bifidobacterium* efficiently consumed 2′-FL and 3′-FL, and that the growth of *Clostridium perfringens* and *E. coli*, which can hardly use fucosylated HMOs, was significantly inhibited by the increase in lactic acid and SCFA concentration [171].

The combination of probiotics and prebiotics, known as synbiotics, has potential synergistic effects that may enhance the functional qualities of infant formulas. Lee *et al.* conducted an evaluation of the safety of a formula containing *Lactobacillus reuteri* (*L. reuteri*), GOS, and FOS in healthy-term newborns. Contemporary infant formula synthesis frequently integrates prebiotics and probiotics to provide a synergistic effect (synbiotics). The mixture of FOS/GOS and *L. reuteri* DSM 17938 demonstrated an elevation in bifidobacteria counts relative to the control formula, which included just probiotics [172]. Alliet *et al.* found same findings, indicating that fecal *Bifidobacterium* counts were markedly elevated in infant formula supplemented with 2′-FL and *L. reuteri* DSM 17938 in comparison to the control group receiving probiotics [173].

### 4.2. Anti-Infective Properties of HMO

By functioning as soluble decoy receptors, HMOs stop infection, whereas bacteria, viruses, fungi, and protozoan parasites attach to glycocalyx to infiltrate hosts. By combining with pathogens, these receptors stop them from attaching to receptors on the surface of epithelial cells, enabling safe passage [174,175].

#### 4.2.1. Bacterial Infections

HMOs protect infants against infectious disorders by serving as prebiotics, which provide beneficial microorganisms a selection edge over pathogens. Symbionts can proliferate and outcompete dangerous intruders through competitive exclusion because pathogenic bacteria are less able to metabolize HMO species [176]. Additionally, organic acids produced by bifidobacterial HMO metabolism produce an acidic environment that inhibits the development of harmful bacteria [177]. By functioning as soluble receptor decoys, HMOs not only indirectly prevent pathogenic entrance but also directly do so. The layer of carbohydrates that covers epithelial cells is called the glycocalyx, and it is made up of glycans that have been linked to proteins or lipids [178]. The glycans that pathogenic bacteria use to adhere to the surface of epithelial cells and the chemical structures of HMOs are comparable [179]. Pathogens and toxic substances that identify and attach to HMOs instead of cell-surface glycans will therefore traverse the gastrointestinal system without inducing illness. OSs may suppress infections by competitive binding to host cell surface receptors [180].

One of the most frequent causes of diarrhea that causes death in newborns appears to be *Campylobacter jejuni* [181]. Because 2′-FL functions as a soluble decoy receptor for *C. jejuni*, it reduces colonization by 80% [182,183]. Enteropathogenic *E. coli* (EPEC), a major cause of baby diarrheal illness, is far less likely to colonize when pre-incubated with a mixture of HMO components [184].

In addition to diminishing pathogen adherence and invasion, HMOs can alter epithelial surface gene expression and stop pathogen development to lessen infection. Genes of Caco-2Bbe gut cells that mediate intestinal epithelial cells’ adherence to *Listeria monocytogenes* would be down-regulated following pre-incubation with HMOs because eIF2 signaling and the unfolded protein response would be activated [185]. With dosages ranging from 1 to 2 mg/L causing up to 96–98% delay, the study discovered that HMOs may influence the development and biofilm formation of Group B *Streptococci* (GBS). The strongest inhibitory capability was demonstrated by LNT and LNDFH-I [186].

#### 4.2.2. Viral Infections

Viral infections pose a substantial risk to public health, especially with the existence of vaccinations for influenza and rotavirus. The presence of evolutionary virus variations and the scarcity of anti-viral medications impede the efficacy of vaccination and long-term therapy. HMOs play a significant role in offering protection against many viral infections. They augment the maturation of the immune system, regulate the Th1/Th2 cytokine response, and provide protection against viral infection by promoting the maturity of epithelial cells and influencing the variety of the microbiome and the growth of commensal gut bacteria. HMOs exert their antiviral effects by emulating the sugar chains of glycoconjugates, which are cell surface carbohydrates that do not adhere to cells [187]. In the early stages of infection, viruses identify sialylated glycoproteins and carbohydrates from the host’s blood group as receptors. Early infection can be avoided by using any carbohydrate that shares structural similarities with these glycans as a receptor decoy. Secretory blood group carbohydrates, including sialylated glycoproteins and fucosylated Lewis antigens, are found in human milk and help ward against viral infections.

HMOs, exhibiting structural similarity with epithelial cell surface glycans, can inhibit the colonization of viral pathogens by acting as soluble decoy receptors or by indirectly obstructing viral adherence through interaction with the epithelial surface [188]. Within the context of the viral infection process, interactions, such as those between carbohydrates and lectin, are very necessary [189]. Pattern recognition receptors are found in the innate immune system, which is responsible for identifying pathogens. On the other hand, viral surface lectins recognize human epithelial cell-surface glycans, which are important for identifying hosts during infection. HMOs have been investigated for their potential to safeguard against viral invasions by imitating the glycans on epithelial cell surfaces. They have immunomodulatory properties, diminishing viral infections in influenza, rotavirus, respiratory syncytial virus, HIV, norovirus, and the condition necrotizing enterocolitis (NEC), which may occur with viral infections [190].

HMOs can enhance newborn immunity against two lethal gastrointestinal diseases caused by rotavirus and norovirus [191]. Mechanical investigations demonstrated that HMOs offer protection against viral infections by imitating receptor sites to obstruct viral entry into host cells [192] and by enhancing immunity through the production of γ-interferon and IL-10 to reduce virulence [193]. Fucosylated and sialylated HMOs diminish rotavirus infection by functioning as decoy receptors, but 3′-SL and 6′-SL more efficiently lower viral infectivity in a pig rotavirus model [191].

As soon as it is ingested, HMO wraps itself around the laryngopharyngeal tract. Specifically, it was predicted that it would reduce the attachment of pathogens to the epithelial cells of the respiratory mucosa at the place of entry [194]. The research investigates the impact of HMOs on the advancement of viral infections and the expression of cytokines. Results indicate that HMOs, when present at concentrations lower than those seen in breast milk, augment innate immunity against respiratory viruses and potentially influence innate immune responses. Immobilized attachments, such as 3′-SL and 6′-SL, impede hemagglutination in influenza viruses, hence obstructing infection [195,196]. Additional Sia residues with an HMO moiety that has been shown to demonstrate a binding affinity toward the influenza virus have been found. According to the findings of a preclinical investigation, 2′-FL might improve the responses to vaccination in mice, hence enhancing the humoral immune response as well as the cellular immunological response. This may have occurred as a result of the compound’s direct influence on the formation of immune cells [197].

HMOs can combat noroviruses by inhibiting their attachment to epithelial tissue surfaces. These compounds imitate blood-active mucin-type *O*-glycans and have the ability to compete with viral glycan receptors. Although our understanding of HMOs is limited, the 2′-FL trisaccharide, a minor but prevalent HMO, efficiently inhibits viral binding [193,198]. Similarly to human noroviruses, sensitivity to certain rotavirus genotypes has been found to be associated with an individual’s blood group status. Selective interaction of rotaviruses with HMOs has been shown in animal experiments. Infant pigs were nourished with HMOs or a combination of them, and then exposed to a strain of pig rotavirus. The length of diarrhea was reduced in piglets that were fed a combination of GOS and FOS. Pigs administered HMOs exhibited a twofold increase in NK cells and a fivefold increase in basophils [193].

#### 4.2.3. Fungal Infection

With systemic candidiasis invasion rates as high as 10% and infant death rates in the early stages of pregnancy at 20%, intestinal fungus has a substantial negative influence on the health of newborns [199].

The study sought to evaluate the efficacy of HMOs in safeguarding human premature intestinal epithelial cells (pIECs) against invasion by *Candida albicans*, a prevalent fungal colonizer in preterm infants. At a physiologic dose of 15 mg/mL, HMO treatment decreased *C. albicans* invasion of pIECs by 14–67%, with a 52% reduction. The delay in hyphal development following yeast inoculation was linked to 30% shorter hyphal lengths. As a result, the total expression level of genes relevant to hyphal formation was reduced by 23 percent. During hyphal induction, HMOs also reduced the quantity of *C. albicans* cells that could attach to pIECs [200].

### 4.3. HMOs Modulating Epithelial Cell Responses

HMOs can have a direct impact on microorganisms and also indirectly by modifying the reactions of host cells. They have been demonstrated to regulate the apoptosis, proliferation, and differentiation of intestinal epithelial cells [201]. Intestinal barrier regeneration occurs by the generation of epithelial cells at the base of the villi, which then migrate into the lumen and undergo differentiation. Enterocytes are responsible for food absorption, in contrast to goblet cells that secrete mucus to defend the lumen against harmful microorganisms [202]. Regulation of epithelial cell proliferation is necessary to prevent excessive stimulation of cell proliferation, which may lead to the development of intestinal cancer [203].

HMOs have been demonstrated to modify gene expression in intestinal epithelial cells, resulting in alterations in the cell surface glycocalyx. In addition to acting as soluble decoy receptors as previously demonstrated, HMOs may also alter the expression of glycocalyx receptors by reprogramming the epithelial cell, hence influencing microbe–host interaction [204]. Neutral HMOs impede the proliferation of intestinal cells by differentiation and/or death, potentially affecting the development of the intestinal barrier. They induce differentiation *in vitro*, enhancing the epithelial barrier’s capacity to process nutrients [205]. The confirmation by Perdijk and colleagues that sialylated HMOs have an impact on intestinal cell homeostasis *in vitro* underscores the advantageous influence of HMOs on epithelial cells [206].

After being fermented by *B. infantis*, HMOs also have indirect effects on the epithelium. According to a research study, *B. infantis* conditioned medium (BCM) improved the expression of junctional adhesion molecule and occludin in either HT-29 or Caco-2Bbe, which can promote intestinal barrier function [207]. Additionally, BCM reinforced the intestinal barrier by up-regulating the production of the claudin-1 protein [208] The BCM may inhibit IL-1b activation to safeguard Caco-2Bbe via the NF-κB pathway as well [209].

The absorption of HMOs by intestinal cells can influence the protein expression of epithelial cells. Acidic OSs are transported by non-specific paracellular channels, whereas neutral OSs transit via paracellular or transcellular routes that involve receptor pathways [210]. Furthermore, the impact of HMOs on gene expression was shown in living organisms, revealing that the addition of sialylated HMOs to rats alters the expression of genes in the intestines and regulates the composition of the intestinal glycome [211].

According to the findings of a study, dietary intervention with 2′-FL increased mice who received an influenza vaccine showed an increase in both their humoral and cellular immune responses. This was achieved by increasing the number of delayed-type hypersensitivity responses, increasing the blood levels of immunoglobulin proliferation that was unique to the vaccination, and increasing the production of CD4+ and CD8+ T-cells in addition to interferon-gamma in the spleen cells of mice that were given 2′-fluorouracil [197].

### 4.4. Other

#### 4.4.1. Brain Development

HMOs are metabolic byproducts that stimulate brain growth, facilitating neuronal communication and synaptogenesis, therefore supplying vital nutrients for brain development and cognitive function [212]. Both developmental and functional activities in the brain are significantly dependent on Sia, which is attached to proteins and glycolipids [213]. Given the significant requirement and abundant concentration of Sia in breast milk, it is essential during early life [214]. Nowadays, several fundamental research models provide evidence that sialyllactoses have an impact on brain and cognitive development. Interfering with cognitive domains, dietary 2′-FL enhances learning and memory in rodents [215]. In addition to the prebiotic function of HMOs and the impact of microbiota on the brain–gut axis, recent studies have demonstrated that fucosylated HMOs, such as 2′-FL, reduce colon motor contractions in an ex vivo model [216].

Research indicates that increased levels of 3′-SL in breast milk in offspring with HMO A-tetrasaccharide (made by mothers with blood type A) are linked to improved development of expressive and receptive language skills [217]. In a study of 485 Malawian breast-fed infants who received FUT2-positive milk, the presence of total sialylated HMOs, particularly the concentration of total fucosylated HMOs, was found to be favorably correlated with language development. Conversely, the structures of non-sialylated and non-fucosylated HMOs exhibited an unfavorable relationship [218]. Similarly, a pilot study with 76 people found a positive connection between breast milk 6′-SL levels at 1 month and composite cognitive score at 18 months. [219].

Gut–brain communication pathways involving gut microbiome metabolites are likely important. Therefore, carefully controlled intervention studies with particular HMOs are needed to prove a causal association in this growing field.

#### 4.4.2. Necrotizing Enterocolitis (NEC)

The severe and frequently fatal intestinal condition known as NEC affects 5–10% of preterm infants with low birth weight, resulting in over 25% mortality. Infants who survive often experience neurological deficits, while breast-fed infants have a reduced likelihood of such problems [220,221]. Despite advancements in infant milk formulas over the last 10–15 years, the disparity in the risk of NEC between newborns fed with formula and those fed with breast milk has remained constant. HMOs continue to enhance the preventive properties of human milk [222].

As previously mentioned, ingested HMO compounds are resistant to gastric acid and intestinal absorption. They enter the small intestine mostly intact and are believed to directly interact with luminal gut microorganisms and intestinal epithelial cells [65]. Kuntz *et al.* showed that HMO causes growth inhibition in intestinal epithelial cells [201,223]. Consistent with this finding, it has been additionally discovered that HMOs promote the development of mucin-2 (Muc2)-producing goblet cells in enteroids generated from humans and in an experimental model of NEC [224]. Human observational studies conducted by Autran *et al.* [225] and Van Niekerk *et al.* [226] have identified a correlation between a low concentration of DSLNT in breast milk and an elevated risk of NEC in premature infants who receive it. Maternal milk fed by infants with NEC had considerably lower DSLNT compared to age-matched controls, as reported by Masi *et al.* [227]. Infants given milk with low DSLNT have a reduced relative abundance of *Bifidobacterium* spp. In addition, Wejryd *et al.* found that mothers of newborns with NEC had reduced levels of both HMO diversity and Lacto-*N*-difucohexaose I (which is exclusively produced by secretor and Lewis-positive women) compared to non-NEC infants [228].

An association between the HMO profile in maternal breast milk and the risk of NEC has generated interest in enhancing the distribution of HMOs to susceptible newborns as a preventative measure. Nevertheless, modifying the HMO profile in maternal breast milk is difficult because of genetic influences and the stage of pregnancy upon delivery. There are several feasible applications for utilizing donated human milk.

#### 4.4.3. HMO and Allergies

Allergic reactions are a significant public health concern in industrialized nations; nevertheless, there is no treatment approach that has been shown to be useful in managing allergic reactions at this time. There is a correlation between allergy disorders and a breakdown in immunological tolerance, a dysbiosis of the microbiota, and an increase in epithelial permeability. The fact that they may be identified during the first month of life raises the idea that these biological systems might be preprogrammed throughout early infancy, which could potentially reduce the likelihood of allergic reactions occurring thereafter. Through their prebiotic qualities and immune-modulating effects, HMOs provide intricately interwoven pathways that modify an individual’s vulnerability to allergies. In this particular setting, there have only been a few preclinical and clinical investigations carried out up until this point [229]. An interventional research study was conducted by Nowak-Wegrzyn and colleagues with the purpose of determining whether or not infants who were allergic to cow’s milk could accept a formula that was substantially hydrolyzed and supplemented with two HMOs. According to the findings of the study, a formula made from whey that contains 2′-FL and LNnT and is clinically hypoallergenic can successfully treat cow’s milk protein allergy in newborns and young children since it contains these ingredients [230]. While working on a β-lactoglobulin-induced milk allergic mouse model, Liu *et al.* recently demonstrated that supplementation with 2′-FL for four weeks decreased the levels of IgE and β-lactoglobulin-specific IgE in the serum and increased the levels of the anti-inflammatory cytokines IL-10, TGF-b, and IFNg in comparison to allergic mice that were not treated with 2′-FL, thereby preventing the development of allergy [231]. HMOs were demonstrated to be effective candidates for the treatment or prevention of allergic symptoms in a mouse model designed to simulate food allergies. In point of fact, allergic mice who were given 2′-FL as a supplement had a reduction in allergy symptoms such as diarrhea and hypothermia, as well as a suppression of protease 1 production from mast cells that was mediated by antigen. This was in comparison to allergic mice that were not given the supplement. A decrease in the passive reaction to cutaneous anaphylaxis was also noticed by the researchers [232].

Differences in supplementation techniques, concentrations, and types of HMOs all contribute to the fact that clinical trials produce inconsistent results, which makes it more difficult to comprehend how HMOs influence allergic diseases. Innovative approaches could provide clarity regarding this matter.

#### 4.4.4. Cardiovascular Diseases

Ongoing study is being conducted in the field of cardiovascular health of preterm infants who are now in the early stages of adulthood. It is possible that early exposure to breast milk might have preventive benefits by halting the pathophysiological changes that occur, which could potentially prevent the development of cardiovascular disease risk in adulthood. An increased appreciation of the possible role that human milk might play in both short-term and long-term cardiovascular health has been developed as a result of the rising understanding of human milk composition. This information may also give insight into the mechanism by which human milk functions as a protective cardiovascular agent [233]. Although it has been demonstrated that HMOs are absorbed by infants and that they play significant roles in the control of immunological and inflammatory responses, it is still unknown whether or not HMOs are particularly crucial for the development of the cardiovascular system and the cardiovascular system in its early stages. It has been demonstrated via research conducted on animals that HMOs have a special role in the promotion of vasodilation, which is mediated by nitric oxide (NO). This may be one of the factors that contribute to their involvement in avoiding NEC. It is therefore possible that the promotion of vasodilation through the mechanism of NO may also help in the preservation of newborn hemodynamics and support the proper development of the cardiovascular system and the heart [234].

It has been shown that breastfeeding has positive effects on the cardiovascular health of mothers, and these advantages may overlap with those that promote cardiovascular development in extremely premature newborns. There is a possibility of a dose–response connection, which would have beneficial impacts on the metabolic health of infants, including the maintenance of lipid homeostasis and glucose breakdown. There is a need for more study in order to test these approaches [235].

## 5. Innovative Approaches to HMO Production: Alternative Sources and Applications

HMOs are now being produced on a big scale and at a relatively low cost, largely due to recent breakthroughs in bioengineering microorganisms that employ basic sugars to produce complex HMOs [236]. Although synthetic, but structurally equivalent, HMOs are now included in initial infant formula formulations, their use beyond the maternal–infant context is now being investigated.

In contrast to milk from farmed animals used for human sustenance, which contains only minimal quantities of OSs [237], human milk has a somewhat substantial number of varied HMOs. For the purpose of bridging the gap in infant nutrition between animal milk-derived breast milk substitutes, several technical approaches can be employed. For instance, it is possible to concentrate the OS-rich portion of animal milk, which can yield certain OSs such as sialyllactoses. However, this method is technically difficult due to the low initial quantities. Conversely, individual HMOs can be synthesized via chemical, enzymatic, or biotechnological methods [238].

Currently, several biotechnological techniques utilizing bacterial fermentation are the most economically feasible technologies [19]. A small number of distinct HMOs are commercially accessible, which are among the most prevalent HMOs contained in breast milk [4]. Most HMOs are present in human milk, but they are less abundant and have less intricate structures in the milk of other mammals [239]. The milk of non-human primates exhibits considerable variation in both the quantity and structural variety of OSs. The reasons behind the extremely distinct OS quantity and composition found in human milk are yet unknown. However, this peculiarity poses significant difficulties in studying the biological significance of HMOs [240].

Technological progress in the isolation and synthesis of HMOs has greatly influenced HMO research, resulting in a greater variety and quantity of accessible HMOs. This has enabled *in vitro* and in vivo scientific investigations, uncovering possible advantages of HMO. The published data have stimulated fresh attention, prompted further resources, and expedited the advancement of HMO separation and synthesis technology (Table 1) [236].

### 5.1. Isolation of OS from Human Milk Donor and Dairy Stream

In the course of research, human milk is commonly isolated for a variety of purposes, including structural characterization, *in vitro* discovery investigations, and the development of analytical standards. There are a variety of biological components that are included in the complex matrix that is human milk. These components include carbohydrates, lipids, proteins, cells, and metabolites. Proteins are precipitated with the use of organic solvents or membrane filtration, and fat is removed from the OS fraction using centrifugation or solvent extraction. [129]. Gel permeation chromatography (GPC), ion-exchange chromatography (IEC), and affinity chromatography (AC) were the methods that were utilized in a number of previous experiments in order to successfully isolate HMOs in trace amounts. In recent years, preparative high-performance liquid chromatography (HPLC) has developed as a valuable method for the extraction of substantial quantities of compounds from natural sources [242].

HMOs separated from human milk have the potential to be consumed by humans in addition to being used for research, particularly by undernourished newborns. For babies who cannot be given their mother’s milk or who require supplements, WHO and UNICEF advocate using donor human milk [241]. The processing of donor milk now places a major emphasis on guaranteeing safety and retaining the nutritional and biological characteristics of the milk. However, further processing has been introduced to boost certain components of human milk in order to provide certain health benefits. For instance, Prolacta Bioscience has developed a cream supplement made from human milk that increases the calorie density of meals, allowing premature children with very low birth weights to be released earlier [243]. The recent finding that whey permeate comprises OSs offers a viable option for the industrial synthesis of OSs similar to those found in human milk. Research indicates that milk OSs traverse the ultrafiltration membranes typically employed in the dairy sector, resulting in their presence in the whey permeate [244].

Human milk-derived HMO products, encompassing particular HMOs or combinations of OSs, obtain a diverse array of HMOs not available elsewhere. Nonetheless, isolation necessitates substantial quantities of donor human milk, characterized by intrinsic variability, which poses significant costs and ethical dilemmas. Consequently, additional sources are required to generate HMOs for human consumption, including dietary or pharmacological components, to address these limitations [241].

The procurement of HMOs is insufficient to fulfill the requirements for interventional clinical investigations, much less for commercialization as ingredients, due to the limited availability of these HMOs and the ethical limits that are associated with their use. It is possible that bovine milk OSs (BMOs) and caprine milk OSs (CMOs), which have structural similarities with HMOs, are capable of performing biological functions that are comparable to one another. It has been found that sialylated OSs generated from human and bovine milk are equally associated with growth augmentation in newborns that are malnourished [245,246].

The characterization of BMOs has been a persistent research focus, although their examination continues to be difficult due to their complexity and low concentration. Recent improvements in analytical technology enable the comprehensive annotation of hitherto uncharacterized OSs, which is essential for assessing the viability of establishing commercial sources for these OSs [247,248]. BMOs comprise essential monosaccharides present in HMOs; however, their quantity is markedly lower, ranging from 0.7 to 1 g/L in bovine colostrum and just negligible levels in mature bovine milk [249]. Nonetheless, its restricted availability renders industrial utilization unfeasible [250]. Given the reduced quantities of mature bovine milk, it is essential to identify techniques for concentrating these key nutritional constituents [236,251,252].

CMOs have gained a lot of interest in recent years because of the fact that goat milk is a key raw material for the dairy industry, is regarded as an appropriate source of protein for newborn formula and possesses larger amounts and diversity of OSs in comparison to bovine milk [253]. CMOs range from 0.06 to 0.35 g/L in mature milk and 0.2 to 0.65 in colostrum. There are 78 CMO compounds, 40 of which have been fully characterized in the literature. Analogous to BMOs, over 80% of CMOs consist of sialylated OSs, which include both Neu5Gc and Neu5Ac sialic acids. The ratio of Neu5-Gc to Neu5Ac in CMOs is considerably greater than that in BMOs [254].

**Figure 6:**
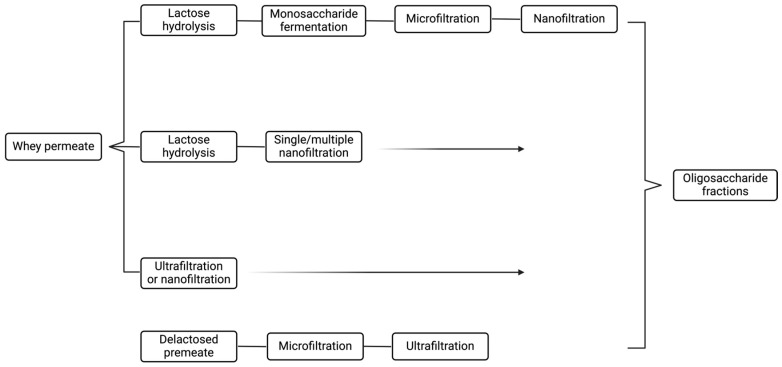
Extraction of oligosaccharides from dairy via membrane technology [241].

Researchers are committed to enhancing BMOs and CMOs through the utilization of membrane-based technologies. This is due to the availability of byproducts that can be collected from dairy processing, which can be used to extract OSs (Figure 6). Finding trace levels of BMOs or CMOs in a lot of Lac is the major issue of the enrichment procedure. Wang and Yu have examined the membrane separation procedure for BMO and CMO enrichment from dairy byproducts, including delactosed permeate, whey, and permeate [255].

The limited availability and compositional variability of OSs in dairy streams and human milk render the extraction of BMOs and CMOs, aside from Lac, a laborious process. A more cost-effective alternative involves the production of OS-enriched products, such as BMO-enriched whey; nevertheless, a cost–benefit analysis is required to identify the optimal manufacturing techniques and industrial uses.

### 5.2. Chemical Synthesis

The manufacture of HMOs has been well explored, as has the topic of carbohydrate chemistry. But compared to other biopolymer syntheses, the synthesis of carbohydrates poses greater difficulties. The biological activity of HMOs depends on precise stereochemical linkages and proper binding. Stereoselective synthesis of glycosidic linkages requires the generation of both monosaccharides with regioselectivity-protected hydroxyl groups and activated glycosyl derivatives [256].

The chemical synthesis of HMOs entails the selective protection of hydroxyl groups, the activation of the glycosyl donor into an electrophilic intermediate, and the removal of protective groups for subsequent processes or the final target molecule. Principal techniques encompass linear synthesis, convergent synthesis, one-pot synthesis, and solid-phase synthesis.

Through the use of a sialyllactosamine derivative and methyl lactoside, Schmidt and Thiem (2010) were able to manufacture LST. The synthesis was carried out in five phases, with each stage utilizing a different protection and deprotection process. The end result was a product yield of 49% [257]. Because of a simplified technique that required just a single purification step, the synthesis of 2′-FL, which is the most common and structurally simpler HMO, was able to be achieved on a kilogram scale. This method was not intended for application in newborn formulas; rather, it is quite beneficial for analytical and testing processes [258].

The labor-intensive and multi-step purification methods result in a poor yield of HMOs from chemical synthesis, ranging from 20% to 55%, which contributes to elevated manufacturing costs. Furthermore, hazardous chemicals utilized in deprotection processes restrict the applicability of chemical synthesis in HMO production [258]. Consequently, chemical synthesis methods are mostly utilized to generate structurally precise HMOs for functional and structure–activity connection investigations. In recent years, chemical synthesis has been employed to provide specific precursors for chemo-enzymatic synthesis.

### 5.3. Enzymatic Synthesis

Enzymatic synthesis offers regioselectivity and stereoselectivity without the need for laborious steps for hydroxyl group protection or deprotection, making it a viable alternative to chemical synthesis.

For the purpose of facilitating the synthesis of glycosidic bonds, glycosyltransferases are responsible for the transfer of an active nucleotide sugar donor to a glycosyl acceptor. In the process of enzymatic synthesis, disaccharide units like Galβ-1,4-Glc, Galβ-1,3-GlcNAc, and Galβ-1,4-GlcNAc play a significant role as acceptors. On the other hand, nucleotide sugars like UDP-Glc, UDP-GlcNAc, UDP-Gal, GDP-Fuc, and CMP-Neu5Ac are the major donors. Identifying appropriate GTs for large-scale manufacturing is difficult due to their chemical properties and poor stability. Researchers investigated the synthesis of nucleotide sugars using multi-enzyme cascades employing high-throughput screening, repetitive-batch-mode synthesis, and enzyme immobilization to minimize expenses [259]. Nonetheless, the restricted stability and elevated expense of nucleotide donors continue to pose considerable obstacles in large-scale manufacturing [260]. GHs can also be employed in glycoside production by promoting trans-glycosylation in opposition to hydrolysis [261]. Engineered GHs with diminished hydrolysis activity have been created to enhance regioselectivity, substrate specificity, and product yield, therefore efficiently catalyzing trans-glycosylation to form HMOs [261,262,263].

On the other hand, for the production of sialylsaccharides, sialyltransferases have been used. By reacting Lac with these enzymes from various sources and donors, simpler structures like 3′-SL and 6′-SL have been produced. For instance, a *Pasteurella dagmatis* sialyltransferase exhibited dual sialidase activity and transferred Sia from κ-casein glycomacropeptide (cGMP), producing both sialylated lactoses in modest amounts [264]. By employing a genetically engineered *Pasteurella dagmatis* enzyme and cytidine-5′-monophospho-*N*-acetylneuraminic acid (CMP-Sia) as a donor, the same products were produced with twice the prior yields [265]. The direct preparation of various HMOs in a milk matrix has been investigated by several researchers. For instance, 3′-SL was obtained when Lac was enzymatically sialylated utilizing two mutant trans-sialidases from the protozoan organism *Trypanosoma rangeli*, which is produced in *Pichia pastoris* [266].

In a different study, Zeuner *et al.* (2019) fused a segment of the amino acid sequence of Clostridium perfringens fucosidase with that of *Bifidobacterium bifidum* to condense 3FL and LNT, achieving a yield of 39% for LNFP II, while diminishing the hydrolytic activity of the hybrid enzyme [267].

This method generates more structurally intricate HMOs than the chemical approach and facilitates the investigation of HMOs’ biological functions in laboratory-scale trials. The range of HMO structures that may be generated by these procedures is limited by the substrate specificity and the availability of glycosidases, despite the fact that enzymatic synthesis has a relatively high efficiency. One of the key focuses is on the development of stable enzymes that provide increased specificities and activity while also reducing the costs of manufacture.

### 5.4. Chemoenzymatic Synthesis

Due to their diversity and complexity, characterizing, quantifying, and biofunctional studies of HMOs continue to be a significant problem. Uniform accessibility of a standardized HMO library is crucial for addressing the long-standing challenges faced by academia for many years [268].

Chemoenzymatic approaches for the production of complex HMOs have been developed as a result of research into isolated enzymes and chemical synthesis methods. This technique, which has been extensively researched for five to ten years, uses the structural properties of HMOs—which consist of just five monomers—to form connections between donor and acceptor molecules at particular locations. Enzymatic regioselectivity and stereoselectivity, thus, aid in avoiding some of the protection and deprotection steps that make such techniques resource- and time-intensive in a normal synthetic approach [269]. Chemoenzymatic methods start with a target substance that has been chemically produced and can be further altered by one or more enzymes linked to glycobiology. If an acceptor is first made, its structure makes it possible to identify certain saccharide units for subsequent enzymatic derivatization [236].

There are currently very few examples of HMO synthesis carried out using a chemoenzymatic method in the literature. Using a sequential one-pot multienzyme (OPME) glycosylation technique, the group led by Xi Chen was able to synthesize LNT, LNnT, and its sialyl and fucosyl derivatives from 3-azidopropyl lactosides that were generated chemically. When it comes to the production of more complex HMOs, the OPME method is a viable technique since it eliminates the need for the time-consuming purification of intermediary phases [270]. For instance, laboratory-scale research has been conducted on the creation of libraries, or sets of combinations of OSs, to produce multiantennary asymmetric HMOs. Prior to being enzymatically altered using various glycosyltransferases, core structures were first chemically created based on the binding of GlcNAc to the galactosyl moiety of Lac via β1–3 or β1–6 linkages. Thus, 60 new structures were created by elongating the original structures by fucosylation and/or sialylation [271].

The variety, production, and purity of HMOs produced in laboratories have all increased thanks to chemoenzymatic techniques. This method enables the creation of large libraries for more structure–function connection research, notwithstanding the difficulty of scaling up for economical commercial applications.

### 5.5. Bioengineering of Microorganisms

Fermentation by microorganisms has been an essential process in the manufacturing of both food and medicinal compounds for a considerable amount of time. For example, microbial fermentation employing natural or modified microorganisms is now used to produce vitamins, amino acids, and antibiotics. Since no bacterium can naturally produce HMOs, metabolic engineering techniques are required to produce HMOs from bacteria.

The development of microbial strains that are capable of synthesizing complex HMOs has garnered the attention of both the academic community and the industrial sector. It is crucial to have competence in enzymatic or chemoenzymatic synthesis in order to simplify enzyme selection and engineering in microbial fermentation. Enzymes are necessary for the creation of metabolic pathways. Nevertheless, several modified strains are unsuitable for commercial production owing to genetic instability and metabolic load, resulting in low-yielding or non-yielding variations during large-scale fermentation [272]. To avert production loss of desirable characteristics, interventions can be implemented during strain formation, such as chromosomal integration, gene deletion, or the building of plasmid addiction systems, rather than relying on single-point mutations [272,273].

Compared to other HMOs, 2′-FL has a comparatively basic structure and is one of the most prevalent HMOs in human milk. For instance, the chosen host for metabolic engineering, *E. coli*, has been modified to generate 2′-FL [100,274,275]. Despite the widespread usage of genetically modified *E. coli* strains, there are certain dangers associated with their use, including the production of certain endotoxins and the requirement in some places for the use of antibiotics. Therefore, using GRAS strains of bacteria and yeast is the best option for fermentation operations. Using a recombinant strain of *Bacillus subtilis* that has been genetically modified to maximize the production of 2′-FL is one such example [276]. Hollands *et al.* (2019) employed fermentation techniques to enhance product secretion into the medium by producing 2′-FL at concentrations of 15 and 24 g/L [277].

Additional HMOs have been synthesized, but with low-to-moderate yields. Despite being a smaller and simpler HMO, 3′-FL has not been as extensively researched as its isomer. Sialylated HMOs, however less prevalent, exhibit several physiologically significant characteristics. Utilizing glycerol and Lac as substrates, 3′-SL was synthesized at a concentration of 25 g/L [278]. This technology involved the overexpression of transferase, epimerase, synthase, and synthetase enzymes in a genetically modified *E. coli*. Utilizing a comparable approach, 6′-SL was synthesized at a concentration of 30 g/L [279].

Upstream and downstream processing make up the manufacturing process, but the most important step is fermentation, which includes product fermentation, seed culture, and production strain growth. Simple sugars, Lac as a substrate for HMO production, glycerol as a carbon source, inorganic salts as nutrients, and certain trace elements as inducers, pH control agents, defoamers, complexation aids, or other processing aids are all used in the fermentation process. The fermentation process keeps on until the desired HMO level is reached. Product concentration (titer), yield, and productivity (rate) are important process performance metrics. The post-fermentation step known as downstream processing, or separation and purification, creates the final high-purity product by separating the fermentation broth’s cell biomass, residual media, carbohydrates, and other byproducts from the target HMO [241].

The microbial synthesis of HMOs can promote their proliferation in the gastrointestinal tract or food, contingent upon the genetic engineering of probiotic or food-fermenting microorganisms, such as yeast or lactic acid bacteria, to produce HMOs.

In conclusion, one of the distinguishing characteristics of HMOs is their distinctive structural characteristics, which include a Lac core, various branching patterns, and a wide variety of monosaccharide compositions, including fucose and sialic acid residues. It is difficult to replicate the detailed structure and function of human milk oligosaccharides HMOs due to the structural variety of these molecules and the delicate biological roles they play. This raises a number of hurdles for the scientific and technical community. The isomeric variety of HMOs and the particular glycosidic connections between monosaccharides, which are frequently regio- and stereospecific, are the sources of the structural complexity of HMOs. As discussed above, it is necessary to have precise enzymatic activities in order to reproduce HMO structures synthetically; however, current approaches are not selective and do not produce the desired results. Despite the fact that glycosyltransferases have shown promise in enzymatic synthesis, there are still substantial challenges to overcome in order to produce enzymes with high specificity. These limitations include cost and scalability [238]. Conversely, replicating the biological roles of HMOs presents significant challenges. HMOs are recognized for their ability to selectively enhance the proliferation of beneficial gut bacteria, such as *B. infantis*, while concurrently inhibiting pathogen attachment to the intestinal epithelium. Non-human OSs, either from plants or animal milk, do not possess specialized glycan structures for targeted effects, and research indicates they fail to successfully replicate the immune-modulatory or anti-infective capabilities of HMOs [8,127]. Furthermore, certain manufactured or naturally sourced OSs have prebiotic properties but may lack the ability to influence the immune system or avert infections, unlike HMOs, resulting in newborns consuming non-HMO OS formulas missing out on the comprehensive health advantages of human milk.

### 5.6. Oligosaccharides in the Milk of Other Mammals

Human and animal milk include a diverse array of OSs, which are of significant interest due to their comparable biological efficacy. Human milk includes a higher amount of OSs compared to non-human mammalian milk. Human milk, notably, displays a distinctive composition of OSs, featuring the highest numbers of discovered (247), structurally characterized (162), and quantified OSs (40) in comparison to other mammalian milks. The concentration of OSs in human milk is significantly greater, ranging from 10 to 100 times that present in non-human mammalian milk [280]. However, OSs are present in farmed animal milk, but in somewhat modest amounts. Among these are 3′-SL, 6′SL, and mostly other neutral non-FucOS such as galactosyllactostoses. In general, their concentration diminishes exponentially from colostrum to fully developed milk [281].

The predominant OS in bovine milk is 3′-SL, which is estimated to have a concentration ranging from 50 to 100 mg L^−1^. In comparison, the amount of 3′-SL in mother’s milk is approximately two times more. Significantly, the concentration of 3′-SL in bovine milk was found to rise from approximately 100 to 700 mg L^−1^ around 2 weeks before childbirth. However, it decreased markedly from around 800 mg L^−1^ in colostrum to around 100 mg L^−1^ by 3 days after childbirth [282]. Albrecht *et al.* (2014) found that camel, porcine, equine, ovine, and bovine milk had 48, 40, 40, 38, and 35 OS structures, respectively. This is a significantly smaller amount than those found in human milk. Other animal OSs share structural components with human milk, but they are less complex and concentrated. In contrast to human milk, domestic animals have a higher quantity of sialylated OSs (80–90%), with 3′-SL and 6′-SL found in nearly all samples [283]. Shi *et al.* found eight unique OSs in human milk and four animal milks: cow, goat, sheep, and camel. Human milk contains these unique eight OSs at concentrations six times more than those in camel milk, twenty times greater than in bovine and caprine milk, and seventy-five times greater than in ovine milk. Bovine, caprine, ovine, camel, and human milk had 30, 42, 32, 34, and 35 oligosaccharides, respectively [284]. Nevertheless, the circumstances are rather distinct with goat milk. Numerical proportions were determined for fucosylated neutral OSs ranging from 15.6% to 18.2%, for non-fucosylated neutral OSs from 7.3% to 18.9%, and for sialylated MOs from 68.2% to 74.1% [285]. The fundamental goal of a related study by Wang *et al.* was to use HPAEC-PAD to assess the Lac content and key OS variations between animal and human milk. Findings indicated that compared to human milk, animal milk had lower amounts of 12 HMOs. The acidic OSs in goat milk were extremely comparable to those in human milk, and only goat milk included 2′-FL, a neutral OS that was plentiful in human milk. Also, donkey milk had a Lac concentration that was most similar to human milk [280]. Zhang *et al.* employed UPLC-QqQ MS and MALDI-TOF MS/MS to identify the eight OS types and their absolute concentrations in the milk of four different mammalian species including human, camel, goat, and bovine. The findings demonstrated that there were differences in the four species’ OS levels, both qualitatively and quantitatively. Camel milk exhibited the greatest quantity of common OSs in comparison to bovine and goat milk. Human milk possesses a greater quantity and absolute concentration of FucOS, while animal milk exhibits a higher number but comparatively lower absolute concentration of sialylated OSs [286].

Goat milk possesses a markedly higher concentration of OSs (gMOS) compared to bovine (bMOS) or sheep (sMOS) milk, in addition to exhibiting a greater structural variety [287]. A research study identified substantial disparities in total gMOS levels between individual goat milk and pooled milk from eight goats, measuring 58.9 mg/L and 178.1 mg/L, respectively, in contrast to human milk and other domesticated dairy species. The osmolarity in goat milk is lower than that in human milk but higher than that in other dairy animals [254]. The concentrations of the main gMOS were highest at the beginning of nursing and decreased over the duration of lactation, which is in line with the findings that were obtained by Claps *et al.* The primary acidic gMOS found in Murciano-Granadina goats was 6′-SL, whereas the amounts of 3′-SL were found to be high in both individual samples and pooled milk from Saanen, Garganica, and Maltese goats [288,289].

Since OS composition varies depending on the species, it has been reported that equine colostrum has a distinct OS content. There have been reports of 43 equine milk OSs (EMOs) to date, of which 12 are acidic and 31 are neutral [290]. Difilippo *et al.* investigated EMOs in equine colostrum with CE-LIF, CE-MS, HILIC-MS, and exoglycosidase degradation methodologies. Differences in EMO prevalence and abundance were identified among and within horse breeds. The predominant OSs in colostrum samples were β 6′ and 3′-galactosyllactose (3′-GL), 3′-SL, and disialyllactose [291].

HMOs have more complexity and variety, particularly in fucosylation and sialylation, relative to other primates. The mean degree of polymerization for HMOs ranges from 7 to 9, signifying bigger and more intricate oligosaccharide structures. The OSs in primate milk demonstrate considerable variation and complexity among species, including humans. Moreover, fucosylation and sialylation levels in primates exhibit considerable variation; chimpanzee milk has 50% fucosylation, gorilla milk has 15%, and siamang milk possesses the highest amounts at 60%, which may be attributable to disparities in microbial interactions and immunological responses. Tao *et al.* conducted a detailed analysis of milk samples from seven monkey species utilizing MS and HPLC-Chip/TOF MS to ascertain the structure and concentration of OSs. The research suggested that monkey milk OSs developed separately as a result of environmental constraints, rather than genetic affinities. Ecological variables such as social group size may also affect their variety, underscoring their adaptive significance in primate nutrition and immunity [292].

The quantification of OSs in animal milk is influenced by genetic diversity and lactation progression, making it difficult to compare research outcomes across different species. Therefore, acquiring supplementary data is crucial for a comprehensive understanding of OSs’ composition in animal milk.

## 6. Oligosaccharides in Infant Formula, Safety Issues, and Clinical Trials

**Figure 7:**
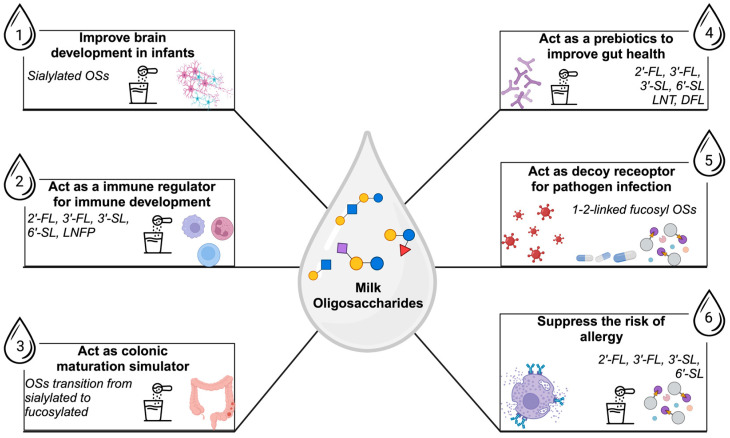
The possible use of OSs in a variety of industries, including pharmaceuticals, dietary supplements, and infant formula [19].

Human milk offers a number of advantages, which can be attributed to the presence of HMOs and bioactive substances. On the other hand, given that not every infant consumes their mother’s milk, it is essential to provide the largest possible nutritional contents (Figure 7). Because it is difficult to synthesize HMOs on an industrial scale, prebiotic compounds such as GOS and FOS that imitate the glycans found in human milk are frequently included in infant formulas [293]. Significant progress has been achieved, and persists, in our comprehension of HMO synthesis; nonetheless, only a limited number of HMOs can now be manufactured in quantities enough for supplementing in newborn formulas (Table 2) [294].

The use of genetically engineered host strains, such as *E. coli*, *Corynebacterium glutamicum*, or *Saccharomyces cerevisiae*, as biological production facilities is now employed in the manufacturing process of HMO supplements for infant formula. The HMOs are produced by the bacteria using basic carbon sources and Lac, and the bacteria then release the product that they have produced into the medium that is used for fermentation. After being refined into a powder that satisfies the needs of certain food market sectors, the product is separated from the supernatant that is produced during cell-free fermentation. Therefore, these HMOs that have been developed can be exploited to improve the nutritional content of foods, such as infant formulas, thereby promoting preclinical and clinical research on the influence that HMOs have on the diet [4,295].

**Table 2:** The highest allowable concentrations of structurally similar synthetic HMOs or combinations of oligosaccharides in infant formulas, measured in grams per liter (g/L) according to The European Food Safety Authority (EFSA) [296].

Oligosaccharides	Structure	Infant Formulas	Infant Follow-On Formulas
2′-FL	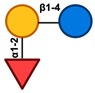	2.4	2.4
2′-FL+DFL	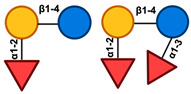	1.6	1.2
3′-SL	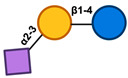	0.2	0.15
6′-SL	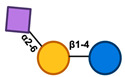	0.4	0.3
LNnT	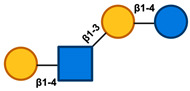	0.6	0.6
LNT	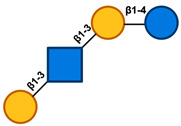	0.8	0.6

In 2017, Puccio *et al.* carried out the initial clinical experiment assessing the effects of infant formula containing two HMOs (2′-FL and LNnT) [297]. Test formula-fed infants had comparable age-appropriate growth, alleviated colic during the initial months, and reduced parental reports of lower respiratory tract infections. Undertaking a subgroup analysis, the immunological role of HMOs was further examined [298].

The Food and Drug Administration (FDA) of the United States of America and the European Food Safety Authority (EFSA) of Europe have both determined that the use of 2′-FL and LNnT as components in infant formula is safe. As a result of developments in bioengineering and manufacturing, 2′-FL has been included in commercial infant formula up until this point. However, in order to evaluate the benefits and drawbacks of including certain HMOs in milk formula in contrast to human milk, a substantial amount of study is necessary [299]. From this point of view, there are a number of clinical studies that have been published in the literature to explore the utilization of HMOs for newborns as well as the health advantages that they provide.

Randomized controlled research was conducted to investigate the gastrointestinal acceptability of a newborn formula that contained 2′-FL and FOS in comparison to a control formula. When the newborn was less than eight days old, the formula was provided, and it was sustained for around one month. The infant showed comparable stool consistency, anthropometric measures, and eating frequency, with occasional spitting up or vomiting. The formula was well tolerated [300]. A research study comparing newborn formulas with and without HMOs revealed that the HMO-containing formula increased *Bifidobacterium* levels, decreased *Escherichia* levels, and elevated Peptostreptococcaceae levels. Nonetheless, no alterations were noted at 12 months, indicating that HMO supplementation may mitigate the negative consequences of not breastfeeding from infancy [301].

The purpose of this study was to investigate the effects of introducing 2′-FL into infant formula on the biomarkers of immunological function. In the case of infants, formulas that contained both 2′-FL and GOS exhibited lower levels of plasma inflammatory cytokines and TNF-α when compared to a control formula that solely contained GOS. There were no discernible changes seen between infants who were breast-fed and those who were fed formulas containing 2′-FL and GOS. Based on the findings, it appears that supplementation with 2′-FL leads to modifications and enhancements in immune development [298]. In their study, Vandenplas and coworkers investigated the effects of a partly fermented infant formula (IF) that included postbiotics, 2′-FL, a specific prebiotic combination consisting of short-chain GOS (scGOS) and long-chain FOS (lcFOS), and milk fat on the development, safety, and tolerance of healthy newborns. The randomized IF groups did not vary statistically significantly in terms of gastrointestinal tolerance or side effects. In healthy-term infants, a partially fermented IF including postbiotics, particular OSs, 2′-FL, and milk fat promotes proper newborn development and is safe and well tolerated [302]. Parschat *et al.* developed the 5HMO-Mix, a blend of five HMOs, to replicate natural concentrations. The composition comprised 52% 2′-fucosyllactose, 13% 3-fucosyllactose, 26% LNT, 4% 3′-SL, and 5% 6′-SL. The research assessed the safety, acceptability, and effects of the 5HMO-Mix formula on growth in healthy neonates. The results indicated no significant variations in the growth of weight, length, or head circumference [303]. The effects of a bovine milk-based formula containing *L. reuteri* (CG), and the same formula with an addition of 1.0 g/L 2′-FL (EG), were compared in a trial including 289 healthy newborns 14 days and younger until they were 6 months old by Alliet *et al.* Weight growth through the age of four months was the main outcome. In addition to being well tolerated and supporting age-appropriate development, infant formula containing *L. reuteri* with 2′-FL may also help to change the gut microbial pattern to more closely resemble that of breast-fed newborns [173].

Lasekan *et al.* performed a research study to evaluate the growth and gastrointestinal tolerance of newborns using a milk-based formula containing a mixture of five HMOs analogous to those present in human milk. The research indicated that including LNT, 3′-SL, 6′-SL, and 2′-FL in human milk improved normal development, tolerance, and safety in healthy term babies. This research seeks to enhance the HMO composition of newborn formulas [304].

On the other hand, the effects of adding 2′-FL to infant formula on growth, adverse event incidence, and infant microbiome—including the expression of microbial genes that metabolize 2′-FL—were investigated by Wallingford *et al.* There were no negative effects or significant impacts of adding 2′-FL on growth in research comparing human milk or baby formula ± 2′FL to breast-fed infants. At baseline, there were no discernible variations in MGS richness or Shannon diversity in fecal samples. After 16 weeks of feeding, formula groups outperformed breast-fed infants in terms of richness and diversity. The GH20 and GH2 families decreased, whereas the GH42 and GH112 families grew in the test formula group. However, breast-fed infants’ microbiomes changed when 2′-FL was added, suggesting that *Bifidobacterium* was involved in the internal metabolism of HMOs [305]. In another clinical study where 2′-FL was used, the findings demonstrated that the study’s formula was safe, well tolerated, and promoted population increase [306].

Vandenplas *et al.* evaluated whether a decreased protein content (2.20 g/100 kcal) and two HMOs in extensively hydrolyzed formula (EHF) promote healthy development in newborns with cow’s milk protein allergy (CMPA). The HMO-supplemented formula significantly reduced the frequency of upper respiratory tract and ear infections in infants with CMPA, as well as the risk of lower respiratory tract and gastrointestinal infections by 30–40% at the age of 12 months. Additionally, the formula significantly decreased the risk of lower respiratory tract infections. In the first year of life, this suggests that there is a preventative impact against infections of the respiratory and ear systems [307]. In a similar clinical trial, Gold *et al.* intended to evaluate whether an amino acid-based formula (AAF) augmented with two HMOs promotes normal development and is well tolerated in infants with CMPA. The study indicated that infants with moderate-to-severe CMPA who received a formula with two HMOs enjoyed acceptable development, with some catch-up growth. The characterization of the gut microbiome revealed initial enrichment of HMO-utilizing bifidobacteria, followed by subsequent enrichment of *Bacteroides* and butyrate-producing taxa. Despite this, there was a reduction in the number of *Proteobacteria*, which is a phylum that is symptomatic of dysbiosis in the gut. In infants, supplementation with 2′-FL and LNnT helped to alleviate the gut microbial dysbiosis condition to some extent [308].

Conversely, the deficiency of diversity in OSs utilized in infant formula has arisen from technological difficulties in acquiring OS structures analogous to those found in human milk. OSs are present in bovine milk, and some of these OSs are structurally the same or almost identical to those found in human milk. The low amounts of these OSs in bovine milk, on the other hand, have impeded efforts to use bovine milk as a source of OSs for infant formula up until very recently [244]. Meli *et al.* investigated baby formulas that included a combination made from whey permeate called BMOs. Two formulas were tested: one with BMOs only, and the other with BMOs plus the probiotics *L. rhamnosus* (LPR) and *B. longum* (Bl999) [309]. On the other hand, given that the majority of infant formulas are made using components derived from bovine milk, it is logical to assume that the amounts of these carbohydrate structures that are consumed by infants who are breast-fed and those who are fed formula are different. Martín-Sosa and colleagues conducted a study to assess the levels of OS-bound sialic acids and major sialyloligosaccharides in human and bovine milk samples acquired at various phases of lactation. The purpose of this study was to discover the differences between the two types of milk and to determine the influence of these changes during the lactation process. According to the findings of the study, the OS content of human milk, cow’s milk, and infant formulas varies. As a consequence, the intake of sialyloligosaccharides is lower in infants who are bottle-fed as opposed to breast-fed infants, and this is true in both qualitative and quantitative aspects [310].

In conclusion, the clinical studies produced a substantial amount of evidence suggesting that HMOs modify the microbiota in the gut and are associated with additional health benefits (Table 3). A critical investigation of the potential health effects that might be caused by increased HMOs has been carried out, particularly with regard to infant formula.

## 7. Current Roadblocks and Future Opportunities

Over the course of time, researchers have identified more than one hundred OSs that are structurally distinct and found in human milk. New technology that allows for quick and high-throughput HMO analysis has made it feasible to conduct large mother–infant observation studies. These studies investigate the correlations between maternal factors and HMO composition, as well as the linkages between HMO composition and a variety of newborn health outcomes. Although there is a general pattern for HMO composition, each woman develops a unique profile of several HMOs at varying concentrations, which might alter during breastfeeding. HMOs are not well digested by the infant, but they act as metabolic substrates for certain gut microorganisms, influencing the gut microbiome’s development as it develops naturally. In connection with this mechanism, evidence has grown that these HMOs protect against infections and other illnesses, help build a breast-fed infant’s brain, and may even improve cognitive function. As antiadhesives, HMOs inhibit different microorganisms from adhering to the infants’ epithelial surfaces, avoiding or lessening infectious illnesses in the gastrointestinal tract and maybe the respiratory and urine tract as well. As antimicrobials, HMOs directly prevent the growth of bacteria and have the ability to act as natural models for the synthesis of novel, much-needed antibiotics. Furthermore, HMOs modify immune cell and epithelial responses, which may impact an infant’s susceptibility to allergies, asthma, and other conditions.

The OSs that are added to the majority of newborn formulas today differ structurally from those found in human milk. It would therefore appear quite unlikely that these non-HMOs could replicate the structure-specific effects of HMOs. It seems that the trend is moving away from the use of non-HMOs and toward the synthesis of OSs that are found in human milk. Subsequent preclinical and clinical investigations will ascertain the efficacy of the now accessible tri- and tetrasaccharides. Gaining further insight into the process of HMO synthesis in the human mammary gland could help direct the development of new technologies that will create a wide range of complex HMO that most nearly mimics the OS composition of human milk. In the long term, breastfeeding continues to be the best option for providing infants with nutrition and care.

## Figures and Tables

**Table 1:** Advantages and challenges of different methods to generate HMOs [56,236,241].

Production Approaches	Advantages	Limitations
Isolation from human milk	-Contains “authentic” HMOs	-The scaling up is considerably restricted due to the limited availability of human donor milk. -Difficult separation and purification
Isolation from dairy streams	-A number of OSs are indistinguishable from those found in human milk.	-Concentrations of OSs in bovine milk are far lower than those in human milk.
	-The scale-up is feasible due to the abundance of enormous volumes derived from dairy streams.	-Bovine milk has very minimal quantities of FucOS, so not all HMOs are found in it.
	-Precursors for chemoenzymatic synthesis can be derived from bovine milk OSs.	-Non-human OSs are present in bovine milk.
Chemical synthesis	-Ability to produce rare HMOs -Highly scalable -High control over structure and purity	-Requires complex, multi-step reactions -Usage of toxic reagents and generates chemical waste -High cost
Enzymatic synthesis	-Certain HMO structures can be produced by precise glycosylation made possible by enzymatic synthesis. -The structural integrity of substrates and products is maintained by the moderate pH and temperature conditions under which enzymes operate. -Environmentally friendly	-Certain enzymes, particularly glycosyltransferases, can be costly to produce and purify. -Enzyme instability -Limited availability of donor substrates -Complexity in producing diverse HMOs
Chemoenzymatic synthesis	-Enables the creation of well-defined structures and HMO libraries, which are particularly useful for studying the correspondences between structure and function. -Easy separation and purification -High conversion rate -High yield	-Challenges in scaling up -Shortage source of enzyme -High cost for glycosyl donor -Difficulty for substrate adaptability -Complex process requiring integration of chemical and enzymatic steps
Bioengineering of microorganisms	-No chemical usage -Sustainable and scalable -Potential for novel HMO synthesis	-Produces a limited set of HMOs at a time. -Utilization of genetically modified organisms. -Risk of contamination and difficulties in purification

**Table 3:** Clinical trials examining the effects of supplementing HMOs in infants.

Study Design	Study Population	Treatment & Dosage	Study Outcomes	References
Randomized, double-blind, multicenter, controlled trial (NCT01808105)	420 Full term, healthy singleton infants (<5 days of age)	-Experiment group 1: 0.2 g/L 2′-FL + 2.2 g/L GOS; Experiment group 2: 1.0 g/L 2′-FL + 1.4 g/L GOS; Control group: 2.4 g/L GOS, and reference group: human milk Duration: 17 weeks	-Infants were provided with formulas supplemented with 2′-FL and designed to have a caloric density comparable to HM. Both growth and 2′-FL uptake were comparable to those of infants fed with HM.	[311]
Randomized, double-blind, multicenter, controlled 3-arm trial	119 Full-term, healthy singleton infants (0–8 days of age)	-Experimental Formula 1 (EF1) and Formula 2 (EF2) were compared with a human milk-fed reference group, with EF1 lacking OSs and EF2 containing scFOS (2 g/L) and 2′-FL (0.2 g/L). Duration: 35 days	-Stool consistency, intake, anthropometric measures, and the percentage of feedings with spit-up or vomiting showed that infants tolerated the experimental formulation containing 2′FL and scFOS.	[300]
Randomized, double-blind, multicenter, controlled trial (NCT03307122)	79 Healthy infants (2 weeks of age (±5 days))	-0.25 g/L 2′-FL + *Bifidobacterium animalis* ssp. *lactis*. %100 whey protein partially hydrolyzed. Duration: 6 weeks	-Well tolerated -The experimental group compared to the control group suggests that 2′FL may boost infant immune system maturation. -The study found that healthy infants fed with sufficient 2′FL human milk had fewer illnesses.	[312]
Non-randomized, open-label, multicenter trial	Healthy term infants 7 days to 2 months at inclusion (*n* = 207)	-1.0 g/L 2′-FL + 0.5 g/L LNnT. Partially hydrolyzed formula (*n* = 66) -Mixed group: HMO formula + BF -BF reference group (*n* = 45) Duration: 8 weeks	-This administration is secure and well-accepted, facilitating normal, age-appropriate development.	[313]
Randomized, double-blind, single-center, controlled trial (NL4627 [NTR4779])	Healthy children between the ages of 1 and 2.5 years (*n* = 461)	-Formula without additives (*n* = 114) -Formula including lactoferrin, immunoglobulins, TGF-β, and milk fat (*n* = 114) Duration: 6 months	-The application is safe, well tolerated, supports normal growth, reduces hard stool days, shortens upper respiratory tract infections, and does not change the gut and nasal microbiome.	[314]
Randomized, double-blind, multicenter, controlled trial (NCT03476889)	Healthy-term newborns under 14 days at inclusion (*n* = 276)	-Study on growth, safety, and tolerance in healthy newborns fed partly fermented infant formula (IF) with postbiotics, 2′-FL, a prebiotic mix of short-chain scGOS and lcFOS, and milk fat. -The Control group has IF with 0.8 g/100 mL scGOS/lcFOS (9:1), whereas the Test Group (IF) has a 26% fermented formula with Lactofidus fermentation postbiotics, 3′-GL, 0.8 g/100 mL scGOS/lcFOS (9:1), 0.1 g/100 mL 2′-FL, and milk fat. Duration: 17 weeks	-This administration is secure and well-accepted, facilitating normal, age-appropriate development.	[302]
Randomized, double-blind, multicenter, controlled trial (NCT03513744)	Healthy-term newborns under 14 days at inclusion (*n* = 341)	-225 participants were randomly assigned to receive either baby formula with 5HMO-Mix (5HMO-Mix) or infant formula without HMOs (IF), while the remaining participants were nursed exclusively. Duration: 4 months	-There was no difference in how 5HMO-Mix was administered in infant formula between the two groups, and healthy term may safely handle 5.75 g/L throughout the first several months of life.	[303]
Randomized, double-blind, multicenter, controlled trial (NCT03090360)	Healthy-term newborns under 14 days at inclusion (*n* = 289)	-Under 14-day-old healthy newborns (*n* = 289) were randomly randomized to receive bovine milk-based formula with L. reuteri DSM 17938 at 1 × 10^7^ CFU/g (CG) or 1.0 g/L 2′-FL (EG) until six months of age. The reference group was unrandomized. Duration: 6 months	-Infant formula with 2′-FL that contains *L. reuteri* promotes development that is appropriate for the child’s age, is well tolerated, and could help change the gut microbial pattern to more closely resemble that of breast-fed infants.	[173]
Randomized, double-blind, multicenter, controlled trial (NCT04105686)	Healthy-term newborns under 14 days at inclusion (*n* = 363)	-Healthy term newborns were evaluated for growth using either human milk (HM; *n* = 104), experimental milk-based formula (EF; *n* = 130) containing five HMOs (5.75 g/L; 2′-FL, 3-FL, LNT, 3′-SL, and 6′-SL), or control milk-based formula (CF; *n* = 129). Duration: 4 months	-The study showed that in healthy-term newborns, the EF comprising five HMOs promoted normal development, GI tolerance, and safe usage.	[304]
Randomized, double-blind, multicenter, controlled trial	Healthy-term newborns under 28 days at inclusion (*n* = 221)	-For 16 weeks, healthy-term newborns were given either human milk or baby formula ± 2′-FL. The microbial community was examined in fecal samples collected at baseline. Duration: 16 weeks	-The incorporation of a physiological concentration of 2′-FL did not influence growth or the occurrence of adverse effects in formula-fed infants, so providing more proof of the safety of this HMO in baby formula. -The incorporation of 2′-FL led to slight alterations in the microbiome, aligning with the characteristics of breast-fed infants and indicating the enhanced metabolic capacity of HMOs by *Bifidobacterium.*	[305]
Non-randomized, multicenter, single-arm trial (NCT03884309)	48 infants under 60 days old who were suspected of having a dietary protein allergy or sensitivity	-A 2-month feeding experiment was undertaken on infants aged 0–60 with suspected food protein allergies, chronic eating intolerance, or conditions requiring severely hydrolyzed formula. The principal outcome was weight maintenance, with a total of 48 newborns completing the trial. Duration: 60 days	-From study day 1 to study day 60, the infants’ weight for age z-scores improved statistically significantly (0.32 ± 0.11, *p* = 0.0078). -The study found the formula safe, well-tolerated, and population-growing.	[306]
Randomized, double-blind, multicenter, controlled trial (NCT03085134)	Non-breast-fed infants aged 0–6 months with (CMPA)	-The test mixture was an extensively hydrolyzed (EHF), 100% whey-based mixture enhanced with 1.0 g/L of 2′-FL and 0.5 g/L of LNnT. A commercially available EHF without an HMO used as the control formula. -The research of 194 infants aged 3.2 to 6 months found that the test formula did not significantly boost daily weight growth compared to the control formula.	-In 12-month-olds with CMPA, HMO-supplemented formula reduced ear, upper respiratory tract, gastrointestinal, and lower respiratory tract infections by 30–40%. This protects against ear and respiratory infections in the first year.	[307]
Non-randomized, open-label, multicenter, single-arm trial (NCT03661736)	Term infants between 1 and 8 months with mild to severe CMPA	-Infants aged 1 to 8 months with moderate-to-severe CMPA participated in research including an AAF enriched with 2′-FL and LNnT) for a duration of 4 months, during which tolerance and safety were evaluated continuously.	-The formula, which was well tolerated and safe, showed a large rise in HMO-utilizing bifidobacteria and elevated fecal SCFAs, while decreasing the quantity of fecal *Proteobacteria*, indicating it partially rectified gut microbial dysbiosis in infants with CMPA.	[308]
Randomized, double-blind, controlled trial (NCT03607942)	Preterm infants (27–33 weeks gestation, birth weight < 1700 g)	-Infants were randomly randomized to receive a 10:1 HMO supplement of 2′ FL and LNnT (0.374 g/kg body weight/day) immediately after delivery (*n* = 43). or an isocaloric placebo (*n* = 43) of Glc (0.140 g/kg/day) till neonatal unit discharge.	-HMO supplementation facilitates early postnatal growth, perhaps enhancing long-term growth and developmental outcomes.	[315]
